# An Intracellular Metabolic Signature as a Potential Donor-Independent Marker of the Osteogenic Differentiation of Adipose Tissue Mesenchymal Stem Cells

**DOI:** 10.3390/cells11233745

**Published:** 2022-11-23

**Authors:** Daniela S. C. Bispo, Catarina S. H. Jesus, Katarzyna Romek, Inês M. C. Marques, Mariana B. Oliveira, João F. Mano, Ana M. Gil

**Affiliations:** Department of Chemistry, CICECO—Aveiro Institute of Materials, University of Aveiro, Campus Universitario de Santiago, 3810-193 Aveiro, Portugal

**Keywords:** adipose tissue mesenchymal stem cells, osteogenic differentiation, cell proliferation, nuclear magnetic resonance (NMR) spectroscopy, metabolomics, endometabolome, polar extracts, donor variability

## Abstract

This paper describes an untargeted NMR metabolomics study to identify potential intracellular donor-dependent and donor-independent metabolic markers of proliferation and osteogenic differentiation of human adipose mesenchymal stem cells (hAMSCs). The hAMSCs of two donors with distinct proliferating/osteogenic characteristics were fully characterized regarding their polar endometabolome during proliferation and osteogenesis. An 18-metabolites signature (including changes in alanine, aspartate, proline, tyrosine, ATP, and ADP, among others) was suggested to be potentially descriptive of cell proliferation, independently of the donor. In addition, a set of 11 metabolites was proposed to compose a possible donor-independent signature of osteogenesis, mostly involving changes in taurine, glutathione, methylguanidine, adenosine, inosine, uridine, and creatine/phosphocreatine, choline/phosphocholine and ethanolamine/phosphocholine ratios. The proposed signatures were validated for a third donor, although they require further validation in a larger donor cohort. We believe that this proof of concept paves the way to exploit metabolic markers to monitor (and potentially predict) cell proliferation and the osteogenic ability of different donors.

## 1. Introduction

Mesenchymal stem cells (MSCs) have become the basis of numerous bioengineering studies due to their ability to differentiate into multiple cell lineages (e.g., adipogenic, chondrogenic and osteogenic) and secrete bioactive factors essential for tissue repair [1,2,3]. However, the clinical value of MSCs is strongly influenced by their intrinsic biological variability (including different donors, tissues of origin or cell subpopulations) and by extrinsic factors (such as non-standardized protocols of isolation, expansion and differentiation) [4,5]. In some cases, seemingly inconsistent results have been reported regarding MSCs’ self-renewal ability, differentiation potential, immunomodulation capacity and morphological characteristics [6,7,8], highlighting the need to monitor/control MSCs heterogeneity and, consequently, improve the reliability of therapeutic outcomes [4].

Inter-donor variability is perhaps one of the major contributions to MSCs heterogeneity, potentially reflecting differences in donor age [9], gender [10], body-mass-index [11], microbiome [12] and general health status [13]. The importance of inter-donor variability has triggered attempts to use data signatures as predictors of differentiation potential [14,15,16]. For instance, certain cell morphological properties [14,15] and surface markers [17] have been proposed to predict MSCs’ quality before their therapeutic application. Furthermore, a combination of donor and cellular characteristics was reported as a possible predictor of successful donor selection for MSC-based bone regeneration [16]. This issue has also been investigated using “omic” approaches that attempted to understand the overall impact of inter-donor heterogeneity on the MSCs genome/epigenome [18,19], transcriptome [20], proteome [21,22] and metabolome [23]. Other efforts focused on gene expression to search for predictive biomarkers of differentiation efficacy [24,25], however, this proved challenging [25] and multiomic approaches were suggested to more accurately address MSCs variability and plasticity, while allowing predictive in silico models to be built [26].

To the best of our knowledge, only a few metabolomic studies of MSCs heterogeneity have been carried out [23,27,28,29,30], employing both targeted and untargeted approaches and mostly using mass spectrometry (MS)-based methods. Metabolomics may be carried out using either MS or nuclear magnetic resonance (NMR) spectroscopy, techniques which provide complementary information [31] and which have proved valuable in determining the detailed metabolic adaptations during cell differentiation [32,33], for instance, to find metabolites with inductive roles in differentiation [34,35,36,37] or aid in the comparison between different MSC donors [23,28,29,30]. For example, an MS-based untargeted study unveiled that human adipose tissue MSCs (hAMSCs) from obese individuals secreted higher amounts of metabolites associated with glycolysis, tricarboxylic acid cycle (TCA) cycle, pentose phosphate pathway (PPP) and polyol pathway while showing decreased proliferation, migration and differentiation abilities [23]. Curiously, obesity affected the metabolome of murine AMSCs differently (namely regarding lipid and amino acid catabolism), indicating that translation from animal models to humans must be treated with caution. Another MS metabolomics report analyzed sphingolipid profiles of human bone marrow MSCs (hBMMSCs) from female and male donors, having suggested body composition and/or hormonal differences as possible contributors to heterogeneity [30]. The age of BMMSCs donors was also shown to affect amino acid and lipid levels, advanced as possibly related to cellular senescence [28,29]. The balance between cell proliferation and senescence may determine the generation of large numbers of MSCs needed for clinical applications [38], and metabolomics has addressed this by comparing metabolic profiles for a range of cell passages [28,39,40,41,42], usually in order to understand for how long MSCs maintain their therapeutic properties (e.g., immunological behaviour and differentiation potentials). In relation to differentiation outcomes, some metabolomic studies have included more than one donor, to identify average metabolic signatures of the differentiation process [26,43,44,45,46,47]; however, to our knowledge, the metabolic differences between MSCs of different donors have not yet been compared specifically. We propose that characterizing and understanding MSCs’ dynamic metabolic behaviour for different donors may provide marker signatures (sets of metabolite variations) with the predictive value of donor “quality” for clinical applications. Inter-donor variability may also be evident in the metabolism of proliferating MSCs (used as controls) and understanding such dependence could help in the definition of specific metabolic markers of differentiation. In the context of osteogenesis, a limited number of metabolomic reports have analysed the stepwise time course of MSCs differentiation, as reviewed recently [33], only a few having considered undifferentiated cells cultured over time as control samples, to the best of our knowledge [35,48,49]. For example, mouse BMMSCs exometabolome changes measured by liquid chromatography (LC)-MS enabled the identification of metabolic biomarkers either (i) specific to control conditions or (ii) common to control and osteogenic conditions (e.g., increases in deoxyuridine and orotidine, possibly linked to cell proliferation) or (iii) specific of osteogenic conditions (e.g., increases in citrate, succinate and glycerol) [48]. Other recent LC-MS reports have proposed osteogenic-specific intracellular variations during 21 d of BMMSCs osteogenesis, such as increases in N6-(L-1,3-dicarboxypropyl)-L-lysine and L-2-aminoadipate [49], whereas metabolic changes in hBMMSCs due to nanovibrational osteogenic stimulation were suggestive of cholesterol sulfate acting as a potential osteoinductor [35].

In this work, untargeted NMR metabolomics was carried out in hAMSCs, for the first time to our knowledge, to search for osteogenesis-specific metabolic markers, which may be considered independent of donor origin and of underlying cell proliferation metabolism. To achieve this, the detailed polar endometabolome adaptations of hAMSCs harvested from two randomly chosen donors, found to differ in proliferating and differentiation extensions, were measured at several timepoints throughout 21 days, under osteogenic and basal (control) conditions. The detailed and holistic nature of NMR metabolomics [33,36,37] enabled different families of hydrophilic metabolites to be identified simultaneously and their time course changes to be determined. The metabolite trajectories observed in common for the two donors, either descriptive of cell proliferation or of osteogenic differentiation, were proposed as potential donor-independent markers of each biological process. Validation of these markers was then attempted for a third donor while recognizing the need for a larger donor cohort for adequate validation to be achieved.

## 2. Materials and Methods

### 2.1. Cell Isolation and Expansion

hAMSCs harvested from two randomly chosen healthy donors (1 and 2) were considered in this study. Adipose tissue from donor 1 was collected through abdominoplasty, under a cooperation agreement between the COMPASS Research Group from CICECO-University of Aveiro and Hospital da Luz, Aveiro, Portugal, upon approval of the hospital Ethics Committee. The patient’s informed consent was obtained under anonymous conditions (unfortunately, characteristics such as gender, age and ethnicity could not be retrieved for this donor). Samples were processed within 24 h after surgery, and hAMSCs isolation was carried out as previously described [50]. The hAMSCs from donor 2 (a 27-year-old African American female) were obtained from a breast reduction operation and purchased from the American Type Culture Collection (Lot 70017032, ATCC PCS-500-011). For donors 1 and 2, both control and osteogenic differentiation assays were performed, as described below. At a later stage of this study, the proposed markers were sought in the cells of a third donor (donor 3). The hAMSCs from donor 3 (a 52-year-old Caucasian female, Lot 63557082) were obtained from a liposuction procedure and purchased from ATCC. For technical reasons, only the osteogenesis assay was performed for donor 3, while a slightly different protocol was followed (namely, different extraction solvent volumes [36], passage 7 compared to 5 and 6 for donors a and 2) and cell proliferation could not be adequately assessed. The cryopreserved hAMSCs from each donor were thawed, plated in culture flasks (T175), expanded and passaged as described before [36,37].

### 2.2. Osteoinduction and Sample Collection

Prior to osteoinduction, hAMSCs were harvested and seeded at a density of 0.5 × 10^6^ cells/flask (cells from donors 1, 2 and 3 at passages 5, 6 and 7, respectively). After reaching nearly 100% confluence under the above-mentioned conditions, the culture medium was exchanged and supplemented with osteogenic differentiation factors, namely, 10 mM *β*-glycerophosphate (Sigma-Aldrich G9422, St. Louis, MO, USA), 50 μg/mL *L*-ascorbic acid (Sigma A0278, St. Louis, MO, USA), and 10 nM dexamethasone (ACROS Organics 230300010, Geel, Belgium). For both donors 1 and 2, parallel control experiments were carried out (i.e., under basal conditions). The medium was exchanged twice a week for 21 days. On days 0, 1, 4, 7, 14, and 21, cells were trypsinized (as previously described [37]) and collected in triplicate (specifically for donor 1, samples were also collected for day 28). For donor 2, two of the three samples of day 21 were discarded due to microbial contamination. Cell suspensions were filtered through 100 μm pore strainers, then centrifuged (300× *g*, 4 °C, 5 min) and rinsed twice in phosphate-buffered saline (PBS) solution, to avoid medium contamination. For donor 3, day 4 samples could not be collected. At least 1 × 10^6^ cells per pellet were used for metabolomics. The above experiments were carried out in 48-well plates to assess cellular proliferation and osteodifferentiation. At each timepoint, plated cells were scraped, rinsed twice with PBS, lysed by osmotic/thermal shock and stored at −80 °C. Media samples (>3 mL) were also collected.

### 2.3. Cellular Proliferation Assessment

Cell lysates were thawed at room temperature (RT), exposed to ultrasounds (10 min) and kept at −20 °C overnight. This was repeated twice to improve DNA extraction. Cell lysates (*n* = 6 samples/timepoint) were centrifuged (450× *g*, 20 °C, 5 min), and supernatants used for double-stranded DNA (dsDNA) quantification (Quant-iT PicoGreen dsDNA assay kit, Molecular Probes, Invitrogen, Eugene, OR, USA). Dye fluorescence was assessed at emission and excitation wavelengths 528 ± 10 nm and 485 ± 10 nm, respectively (microplate reader, Synergy HTX, BioTek Instruments, Winooski, VT, USA). dsDNA concentrations were calculated using a calibration curve in the 0.0−2.0 μg/mL concentration range.

### 2.4. Osteogenic Differentiation Assessment

The activity of alkaline phosphatase (ALP, an early marker of osteogenic differentiation [51]) was assessed by the p-nitrophenol assay, in which colourless p-nitrophenyl phosphate (pNPP) is hydrolyzed by ALP to originate yellow p-nitrophenol (pNP). In a 96-well plate, 60 μL of diethanolamine buffer solution at pH 9.8 (containing 2 mg/mL pNPP) were added to 20 μL of cell lysates collected at days 4, 7, 14 and 21 (*n* = 6 samples per timepoint). The mixture was incubated at 37 °C for approximately 1 h and then 80 μL of 3 M NaOH (in MiliQ water) was added. pNP absorbance at 450 nm was measured (microplate reader, Synergie HTX, BioTek Instruments, Winooski, VT, USA) and ALP activity was calculated using a pNP calibration curve (0.0−0.5 mM). For calcium quantification and determination of osteocalcin (OCN, a late marker of osteogenic differentiation [51]) protocols were followed as described previously [36,37]. ALP activity, calcium concentrations and OCN secretion were normalized by total dsDNA.

### 2.5. Metabolite Extraction and NMR Spectroscopy

Intracellular metabolites were extracted using a methanol-chloroform-water method described elsewhere [52]. Briefly, cell pellets were resuspended in 1 mL of a cold solution of methanol (Honeywell Riedel-de-Haen 14262, Seelze, Germany) and Milli-Q water (4:1), transferred to glass tubes containing 150 mg of glass beads (ø = 0.5 mm), and vortexed for 2 min (2500 rpm, RT). Cold chloroform (400 μL, Honeywell Riedel-de-Haen 650471, Seelze, Germany) was added to each sample (vortexed for 2 min, 2500 rpm, RT), followed by 400 μL of cold chloroform and 360 μL of cold Milli-Q water. The mixture was vortexed for 2 min (2500 rpm, RT). After 10 min at −20 °C, samples were centrifuged (2000× *g*, 20 min, RT), and polar and lipophilic phases were separated. Polar extracts were dried under vacuum and stored at −80 °C until analysis (only polar extracts were analyzed in this work). Importantly, the above conditions constituted an improved protocol in relation to that used for donor 3 cells, reported earlier [36,37]. Polar extracts were resuspended in 650 μL of 100 mM phosphate buffer (pH 7.4), in D_2_O (99.9% deuterium, Eurisotop D216) containing 0.1 mM 3-(trimethylsilyl)-propionic-2,2,3,3-d4 acid (TSP, in D_2_O, Sigma-Aldrich 293040, St. Louis, MO, USA) for chemical shift referencing. After vortexing, 550 μL were transferred to 5 mm NMR tubes. NMR spectra were recorded on a Bruker Avance III spectrometer operating at 500.13 MHz for ^1^H (298 K). Standard unidimensional spectra were acquired with the noesypr1d pulse sequence under the following conditions: 7002.801 Hz spectral width, 32 k data points, 2.3 s acquisition time, 4 s relaxation delay (d1) and 512 scans. Each free induction decay was zero-filled to 64 k points, multiplied by a 0.3 Hz exponential line-broadening function and Fourier transformed. Spectra were manually phased and baseline corrected. As described before [36,37], peaks have been assigned with the aid of two-dimensional ^1^H-^1^H total correlation (TOCSY) and ^1^H-^13^C heteronuclear single quantum correlation (HSQC) spectra, spiking experiments, the literature and spectral databases, such as the Bruker BBIOREFCODE AMIX database, the human metabolome database (HMDB) and Chenomx NMR Suite (Chenomx Inc., Edmonton, AB, Canada). Statistical total spectroscopy (STOCSY) [53] was used to attempt the assignment of new resonances.

### 2.6. Statistical Analysis

Multivariate analysis was applied to the full resolution ^1^H NMR spectra using SIMCA-P 11.5 (Umetrics, Umeå, Sweden), upon exclusion of water (5.11−4.69 ppm) and TSP (0.30−0.00 ppm) spectral regions from the matrices. Due to possible contamination, methanol (3.38−3.34 ppm) and ethanol (3.68−3.63 and 1.21−1.17 ppm) regions were also excluded. Spectra were aligned using recursive segment-wise peak alignment [54], to minimize chemical shift variations, and normalized to the total spectral area, to account for sample concentration (i.e., cell numbers) differences. Principal component analysis (PCA, unsupervised analysis used to reveal intrinsic clusters and outliers within the data set) and partial-least-squares discriminant analysis (PLS-DA, supervised analysis to maximize class discrimination) were performed after centred scaling spectra [55]. Multivariate and univariate statistical analysis and peak integration were carried out as described elsewhere [36,37]. 

## 3. Results

### 3.1. Biochemical Evaluation of Donors 1 and 2

hAMSCs cultured in basal medium (controls) presented overall similar dsDNA patterns (indicative of cell proliferation) to those observed in osteogenic media until day 14 (Figure S1A), except for day 7 where donor 2 showed a relative dsDNA decrease. While proliferation remained high for controls after 21 days, the process stagnated when under osteogenic conditions for both donors, consistent with a previous report of human and rat BMMSCs proliferation, while undergoing osteogenic differentiation triggered by dexamethasone [56,57]. Notably, generally higher cell proliferation extension seemed to characterize donor 1 compared to donor 2, in both culture conditions (Figure S1A). In differentiating cells, an increase in normalized ALP levels was observed up to day 21 for donor 1 (Figure S1B), as expected [58]. For donor 2, ALP values remained constant for the whole 21-day period, with relatively higher levels in osteogenic conditions compared to basal media. The significantly higher ALP values observed for donor 1, for days 14 and 21, are indicative of more extensive mineralization and in vivo bone formation [59]. Normalized OCN levels increased significantly at day 21 for both donors undergoing osteogenesis, compared to controls (Figure S1C), with a tendency of donor 2 for high OCN levels at day 14 (compared to day 21), irrespective of culture conditions. Total calcium quantification (Figure S1D) informs on (i) intracellular calcium, (ii) free calcium ions in the extracellular matrix (ECM), and (iii) calcium ions associated with insoluble mineral deposition (e.g., calcium phosphates). The normalized overall calcium content profile (Figure S1D) showed relatively higher values at day 7 for basal conditions for both donors, possibly reflecting high intracellular calcium content [60]. While calcium levels decreased in basal conditions throughout days 7 to 21 for both donors, in osteogenic conditions a significant increase in calcium content was observed for donor 1 at day 21, while a smaller non-significant increase was noted for donor 2. This seems to reflect a more extensive deposition of calcium phosphate nodules for donor 1, consistently with the higher osteogenesis extension shown by ALP levels. Indeed, an increase in total calcium content (including insoluble forms at pH 7) has been reported for hAMSCs undergoing osteogenic differentiation [58], while calcium stagnation has been interpreted as reflecting stable intracellular calcium levels [60], thus corroborating the lower mineralization extension characterizing donor 2. 

### 3.2. NMR Metabolomics of Cell Proliferation

**Figure 1:**
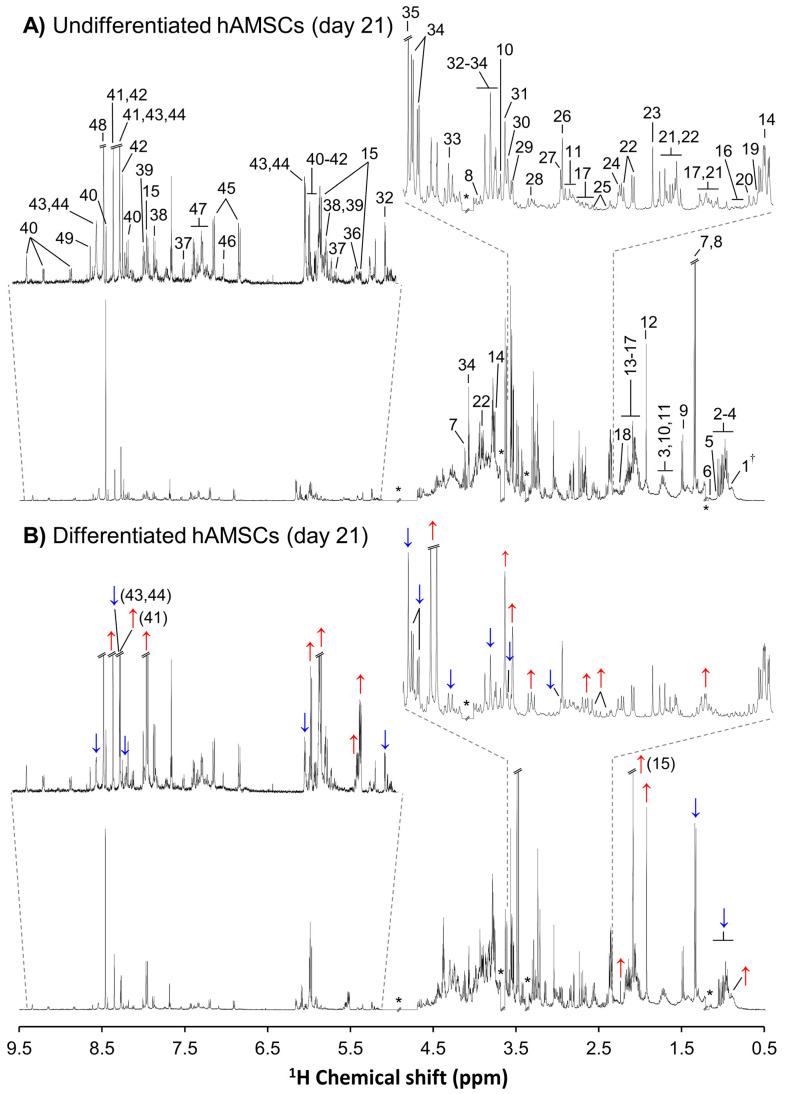
Average ^1^H NMR spectra of polar extracts obtained from (**A**) undifferentiated and (**B**) osteodifferentiated hAMSC of donor 1 at day 21. Apparent metabolic variations with differentiation are identified in (**B**) with blue and red arrows indicating decreases and increases, respectively. †, resonances not observed in day 0 samples; *, excluded spectral regions. Peak assignments: 1. pantothenate, 2. isoleucine, 3. leucine, 4. valine, 5. propionate, 6. propylene glycol, 7. lactate, 8. threonine, 9. alanine, 10. arginine, 11. lysine, 12. acetate, 13. proline, 14. glutamate, 15. uridine diphospho-*N*-acetylglucosamine (UDP-GlcNAc), 16. glutamine, 17. glutathione (reduced) (GSH), 18. acetone, 19. pyruvate, 20. succinate, 21. citrate, 22. aspartate, 23. dimethylamine (DMA), 24. methylguanidine, 25. asparagine, 26. creatine (Cr), 27. phosphocreatine (PCr), 28. ethanolamine, 29. choline, 30. phosphocholine (PCho), 31. glycerophosphocholine (GPC), 32. glucose, 33. taurine, 34. *m*-inositol (*m*-ino), 35. glycine, 36. uridine diphospho-*N*-acetylgalactosamine (UDP-GalNAc), 37. uracil, 38. uridine, 39. guanosine, 40. nicotinamide adenine dinucleotide (NAD^+^), 41. adenosine, 42. inosine, 43. adenosine diphosphate (ADP), 44. adenosine triphosphate (ATP), 45. tyrosine, 46. histidine, 47. phenylalanine, 48. formate, 49. adenosine monophosphate (AMP).

**Figure 2:**
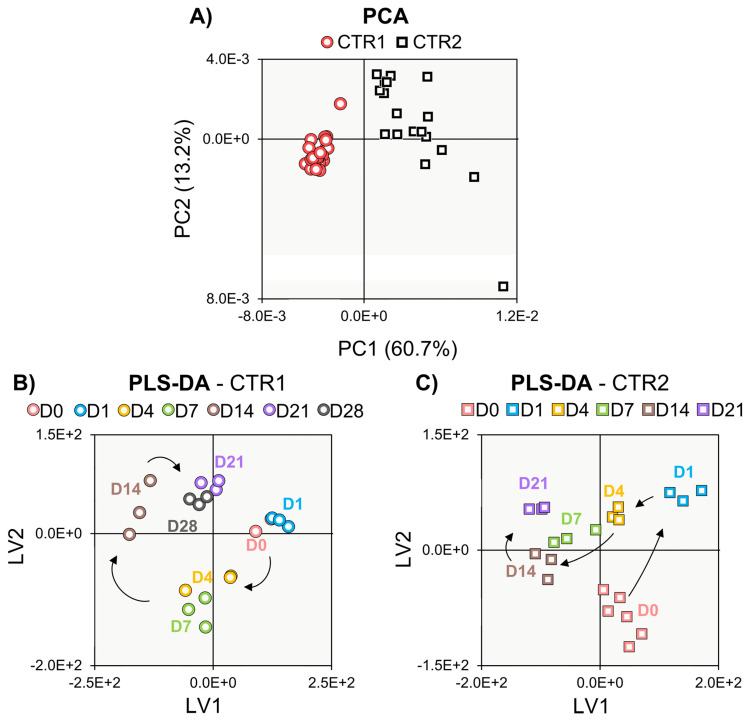
Multivariate analysis of ^1^H NMR spectra of polar extracts of proliferating hAMSCs of donors 1 (circles) and 2 (squares). Scores scatter plots of (**A**) PCA for donors 1 (red) and 2 (black), and PLS-DA for donors 1 (**B**) and 2 (**C**). CTRi, control hAMSCs for donor i; Di, day i; LV, latent variables; PC, principal component.

The average (of 3 replicas) ^1^H NMR spectra of the polar extracts of hAMSCs from donor 1 (either undifferentiated or differentiated, Figure 1A and 1B, respectively) showed high spectral complexity, which translated into the identification of ca. 50 intracellular metabolites considering all samples (Table S1). The peak assignments achieved indicate that NMR spectroscopy provides, in a single record, information on the presence of several compound families (amino acids and derivatives, membrane precursors, nucleotides and derivatives, and organic acids, among others), a feature well suited to untargeted metabolomics. The general spectral profile changed significantly when hAMSCs underwent differentiation (illustrated for day 21 in Figure 1B), although apparent peak changes require validation through statistics, as shown below. This level of compositional information will provide detailed information on the dynamic metabolic adaptations accompanying cell proliferation (controls) and osteogenic differentiation. Regarding undifferentiated cells, clear separation between donors 1 and 2 is seen in PCA (Figure 2A), across the 21-day period. PCA of donor 1 controls alone (CTR1) (Figure S2A) suggested a metabolic change from days 1–7 to days 14–21 (with day 28 samples harvested only for donor 1, closely positioned to those of day 21, i.e., characterized by similar intracellular metabolic profiles). On the other hand, donor 2 controls (CTR2) exhibited larger sample dispersion, as also confirmed by the individual time course representation in PCA (Figure S2B).

**Figure 3:**
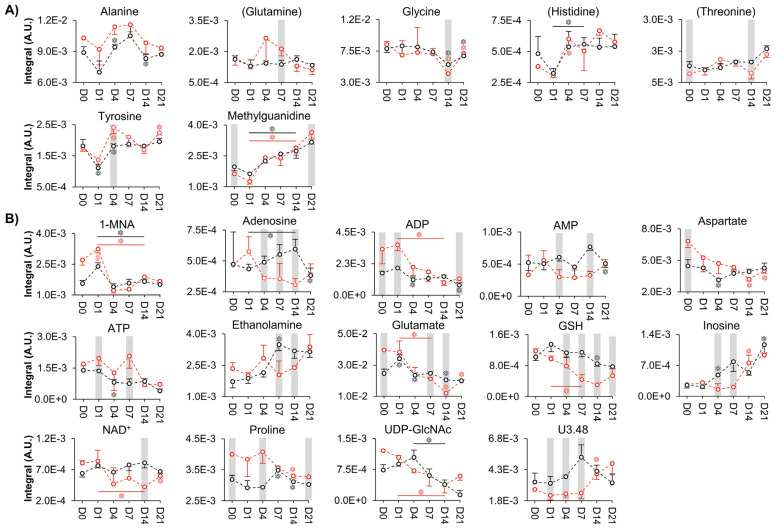
Donor-independent metabolic variations specific to hAMSCs proliferation. The line graphs display the normalized integrals of metabolites from donor 1 (dashed black lines) and 2 (dashed red lines) that (**A**) vary similarly throughout the 21 days or (**B**) converge at the end. Metabolite names in brackets identify compounds with peaks close to the noise level. 1-MNA, 1-methyl-nicotinamide; ADP, adenosine diphosphate; AMP, adenosine monophosphate; ATP, adenosine triphosphate; GSH, glutathione (reduced); NAD^+^, nicotinamide adenine dinucleotide; Uδ, unassigned signal at *δ* chemical shift; UDP-GlcNAc, uridine diphospho-*N*-acetylglucosamine. Symbols (represented in the same colour as the corresponding line): *, Wilcoxon Rank-sum test *p*-value < 0.05 compared to the previous timepoint (shown only in cases where spectral confirmation was observed); #, relevant variations compared to the previous timepoint exclusively based on visual inspection (applicable when *n* < 3 samples, i.e., day 0 from donor 1). Grey bars highlight statistically significant differences (*p-*value < 0.05) for each timepoint between donors (except for day 0, in which the grey bars refer to relevant variations exclusively based on visual inspection because *n* = 2 for donor 1).

**Figure 4:**
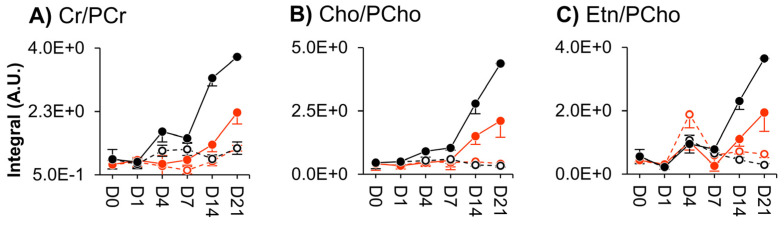
Metabolic ratios as donor-independent markers specific to hAMSCs osteodifferentiation (solid lines) in comparison with their control counterparts (dashed lines); (**A**) Cr/PCr, (**B**) Cho/PCho and (**C**) Etn/PCho for donors 1 (in black) and 2 (in red).

Supervised multivariate analysis through PLS-DA lead to better-defined time course evolutions for both donors (Figure 2B,C), although the corresponding trajectories were clearly different. This shows that cell metabolism dynamics accompanying cell proliferation seem to be, at least in part, donor-dependent. Stepwise variations in peak integrals, expressed in effect size (heatmap in Figure S3), revealed that, for both donors, the levels of many compounds change before day 4 (particularly, for amino acids), whereas from day 4 to day 7 only membrane precursors see their levels changed (increased), except for a proline increase for donor 2 (Figure S3). Direct comparison of day 0 (although with only 2 replicas) and day 21 (right columns, Figure S3) indicated that on day 21, most amino acid levels in donor 2 (slightly less proliferating) return to initial day 0 levels (contrary to donor 1), this levelling off beginning to occur after day 14. For both donors, other metabolite families exhibited several persisting changes, thus contributing to distinct net proliferation signatures. Despite these differences, metabolite trajectory graphs unveiled a set of similar metabolic features of cell proliferation for the two donors (Figure 3). These include almost overlapping evolutions for alanine, glutamine, glycine, histidine, threonine, tyrosine and amino acid derivative methylguanidine (Figure 3A). Other metabolites showed distinct trajectories at some point in time but finally converged to similar values for both donors, generally between days 7 and 21 (Figure 3B). This is the case for aspartate, glutamate, proline, reduced glutathione (GSH), adenosine, adenosine monophosphate (AMP), adenosine diphosphate (ADP), adenosine triphosphate (ATP), inosine, nicotinamide adenine dinucleotide (NAD^+^), uridine diphospho-*N*-acetylglucosamine (UDP-GlcNAc), ethanolamine (Etn), 1-methylnicotinamide (1-MNA) and the unassigned resonance at *δ* 3.48. We hypothesize that the trajectories followed by these 21 metabolites (or rather, 18 metabolites, considering that histidine, glutamine and threonine peaks are very close to the noise level, names in brackets in Figure 3) may characterize cell proliferation, independently of the nature of the donor, although confirmation of this in additional donors is necessary. On the other hand, marked donor-dependent metabolite changes with cell proliferation were noted in 17 metabolites (Figure S4), namely, the three branched-chain amino acids (BCAAs: leucine, isoleucine and valine), phenylalanine and taurine; creatine (Cr) and phosphocreatine (PCr); choline (Cho), glycerophosphocholine (GPC) and phosphocholine (PCho); acetate, formate, lactate and succinate; glucose, glycerol and *m*-inositol. All the above adopted significantly distinct levels at least at day 21 and, many, along parts of the trajectories. Interestingly, although Cr and PCr varied differently for both donors, their ratio was maintained constant during proliferation and overlaps for the two donors (dashed lines in Figure 4A). The same applies to Cho/PCho and Etn/PCho ratios (Figure 4B,C), importantly showing that these metabolite ratios are donor-independent. Furthermore, no significant inter-donor differences were found for nucleotides or their derivatives (note the lack of these metabolites in Figure S4), suggesting that nucleotide metabolism in proliferating hAMSCs is well regulated by different donors, to achieve similar levels at the end of the 21-day period (Figure 3). This is also expressed by a constant adenosine/inosine ratio (not shown), although the corresponding peaks were close to the spectral noise level. 

### 3.3. NMR Metabolomics of Osteogenic Differentiation

**Figure 5:**
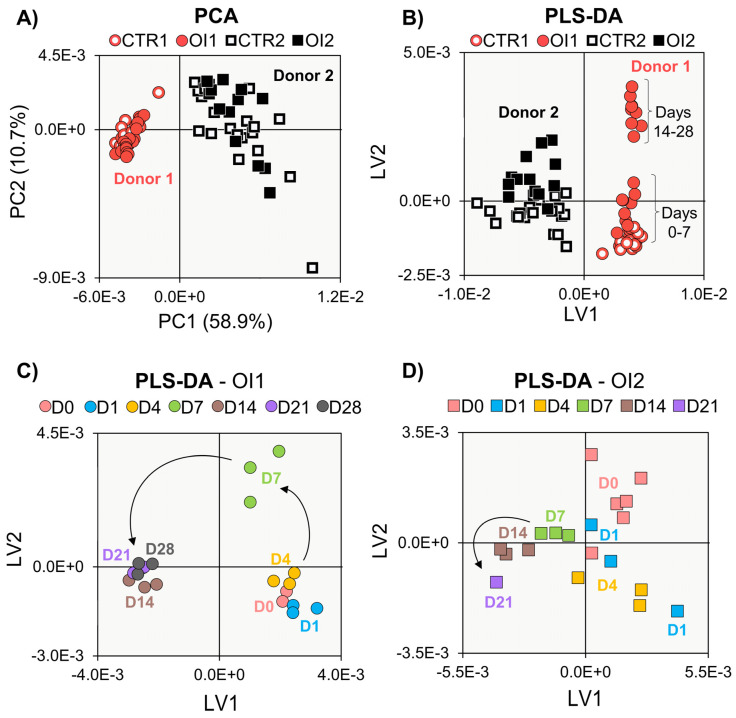
Multivariate analysis of ^1^H NMR spectra of polar extracts of osteodifferentiating hAMSCs from different donors. (**A**) PCA of osteodifferentiating hAMSCs (filled symbols) and their control counterparts (open symbols) for donors 1 (red circles) and 2 (black squares). PLS-DA of (**B**) osteodifferentiating and control hAMSCs from donors 1 and 2, and individual time course differentiations for (**C**) donor 1 and (**D**) donor 2. As day 0 samples were collected immediately prior to osteoinduction, they are considered controls in all models. CTRi, proliferating (control) hAMSCs from donor i; Di, day i; LV, latent variables; OIi, osteoinduced hAMSCs from donor i; PC, principal component.

When osteogenic differentiation was triggered, the distinct donors formed separate groups in PCA (full symbols in Figure 5A), which differed in time course dispersion. Individual PCA trajectories (Figure S2C,D) confirmed this, and PLS-DA clarified that donor 1 differentiating cells show a metabolic switch between days 7 and 14 (Figure 5B), contrary to donor 2. The individual PLS-DA model obtained for donor 1 (Figure 5C) further unveiled a clearer metabolic trajectory of osteogenesis, compared to donor 2 (Figure 5D), running from days 0, 1, 4, to day 7 (isolated from all remaining days), and then to days 14, 21 and 28 (the latter only analyzed for donor 1). Stepwise metabolite variations, for controls and differentiating cells, are shown in heatmaps for both donors (Figure S5), and all metabolite trajectories are represented in Figures S6–S9. Considering the heatmaps, donor 2 showed significant early changes (day 0 to day 1) in amino acids and membrane precursors, mostly absent in donor 1 and mainly due to proliferation (as control and osteogenic signatures almost coincide). Although both donors show osteogenic impact (differences between controls and osteogenic conditions) on all compound families from day 1 to day 14, the endometabolome tended to subsequently stabilize (see blank rectangles in day 14 to day 21 transition, for both donors, Figure S5). 

This stabilization of metabolite levels during osteogenesis seemed clearer for donor 2 (the only exceptions being changes in PCr and lactate), with amino acid levels at day 21 approaching those at day 0 (blank rectangles in the right column in Figure S5). Given that donor 2 undergoes less extensive osteogenesis (and also less proliferation) compared to donor 1 (Figure S1), this suggests that the degree of amino acid changes may not only reflect the proliferation degree (as mentioned above) but also osteogenesis extent. Cr and PCr metabolism is an important feature where changes persist in both donors, upon osteogenesis (Figure S5). These reflected increasing Cr/PCr ratios after day 7 (Figure 4A) due to a steady PCr decrease, overlapping for both donors (Figure S7D, right). The smaller increase observed for donor 1 (more osteogenic and more proliferating) is due to lower levels of creatine (Figure S7A, right). Membrane precursor signatures were also active features in both donors (Figure S5) and Cho/PCho and Etn/PCho ratios (Figure 4B,C) increased after day 14 for both donors due to increasing Cho and Etn (Figure S9A,B) and concomitant PCho decrease (Figure S9D). Again, these ratio increases were less pronounced for donor 1 and this was due to lower levels of Cho and Etn in the more osteogenic donor (Figure S9A,B). Regarding nucleotides and derivatives, the adenosine/inosine ratio (not shown) also showed an increasing tendency in osteogenic conditions, although donor comparison is hindered by the low signal-to-noise of the corresponding resonances. 

**Figure 6:**
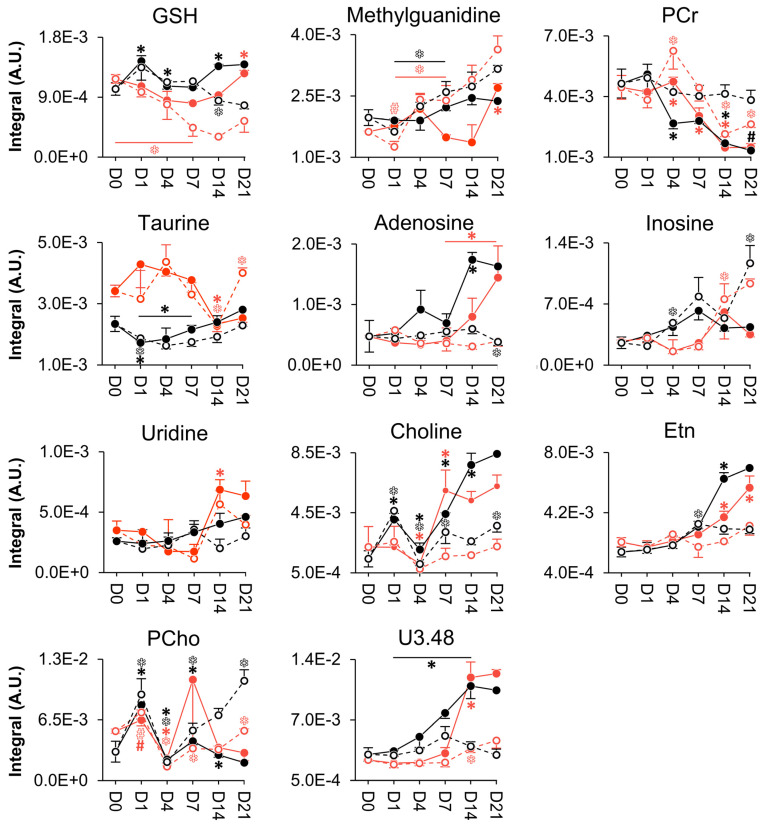
Donor-independent metabolic variations specific to hAMSCs osteodifferentiation (solid lines) in comparison with their control counterparts (dashed lines). Line graphs are defined as described in the caption of Figure 3. Di, day i; GSH, glutathione (reduced); PCr, phosphocreatine; Etn, ethanolamine; PCho, phosphocholine; Uδ, unassigned signal at chemical shift δ. *, Wilcoxon Rank-sum test *p-*value < 0.05 compared to the previous timepoint (shown only in cases where spectral confirmation was observed); #, relevant variations compared to the previous timepoint exclusively based on visual inspection (applicable when *n* < 3 samples, i.e., day 0 from donor 1 and osteogenic day 21 from donor 2). All symbols are represented in the same colour as the corresponding line, with open and filled symbols for proliferating and differentiating cells, respectively.

In order to propose possible osteogenic-specific donor-independent metabolites (although only based on 2 donors and, thus, requiring confirmation in additional donors), selected metabolite trajectories were identified when meeting the following two conditions simultaneously: (i) specificity for osteogenic differentiation (i.e., metabolite trajectories differing from proliferation evolution, at least in part), and (ii) apparent donor-independence (i.e., similar trajectories for donors 1 and 2, at least in the final days of the differentiation process). We propose that 11 metabolites may serve as candidates for osteogenic-specific and donor-independent markers (Figure 6): (1) taurine, PCr (and, in particular, the Cr/PCr ratio), GSH, and methylguanidine; (2) adenosine, inosine (potentially expressed as adenosine/inosine ratio, although the low abundance of these compounds hinders definite comparison) and uridine; (3) Cho, Etn and PCho (also expressed as the corresponding ratios); and (4) still unassigned resonance at *δ* 3.48 (found to correlate to membrane precursors and adenosine through STOCSY).

**Figure 7:**
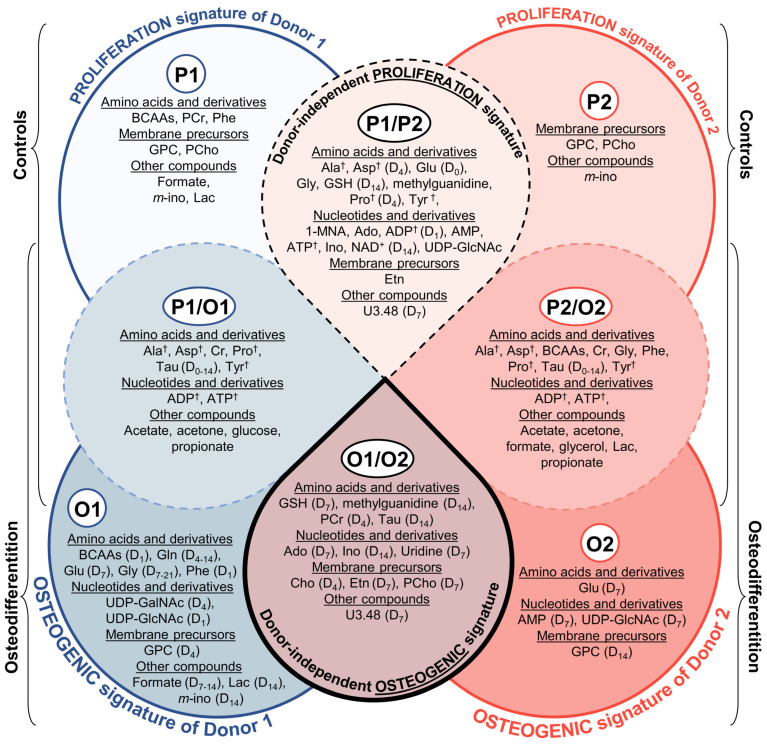
Schematic representation of metabolites proposed to compose donor-dependent and donor-independent signatures of MSCs proliferation and osteodifferentiation. Donor 1 (blue) and donor 2 (red) individual metabolic signatures, exclusive of proliferation and osteodifferentiation are shown in sections Pi and Oi (where i is the donor number), respectively. Metabolites changing in common to proliferation and osteodifferentiation are situated in overlapped sections Pi/Oi (i: donor number). Proliferation-specific and osteodifferentiation-specific metabolic signatures (mutually exclusive) shared by both donors are listed in the overlapped sections P1/P2 and O1/O2, respectively. Each of the overlapped sections P1/P2, P1/O1 and P2/O2 include metabolites that (i) vary similarly throughout the 21 days (metabolites without any time specification), (ii) converge between days i and j (D_i–j_) or (iii) converge after day i (D_i_). Furthermore, metabolites in sections O1, O2 and O1/O2 are followed by the indication of the day (D_i_) or time period (D_i–j_) after/during which the metabolites are considered osteospecific. Importantly, metabolites highlighted with † appear in more than one section but follow similar time course trajectories, whereas repeated metabolites without the † indication vary differently in each section. Compound abbreviations as defined in the caption of Figure 1.

Figure 7 summarizes the results presented above, with sections P1 and P2 (for donors 1 and 2, respectively) including the metabolites seen to vary only in their control samples, thus composing proliferation signatures specific of each donor. Note that GPC, PCho, and *m*-inositol feature in both P1 and P2 but lack the symbol † (see caption of Figure 7), which means that these metabolites follow distinct trajectories in each proliferating donor (Figure S9). In addition, the P1/O1 section includes a set of metabolites which vary both in controls (P1) and osteogenic differentiation (O1) of donor 1, and similarly for section P2/O2 corresponding to donor 2. Within these sections, alanine, aspartate, proline, tyrosine, ADP and ATP follow similar trajectories for the two donors († in Figure 7), both in controls and in osteogenic differentiation. This indicates that the levels of these 6 metabolites mainly robustly reflect cell proliferation, not being significantly affected by the osteogenic process and, thus, indicating that they may help to monitor cell proliferation, even in the presence of osteogenesis. These 6 metabolites are part of the above-identified 18-metabolite signature characteristic of controls for both donors 1 and 2 (P1/P2 section in Figure 7) and thus proposed to be a donor-independent cell proliferation marker. In addition, donor 1 seems to be characterized by a metabolic signature of its own when hAMSCs undergo osteogenic differentiation (section O1), which differs from that of donor 2 (section O2) (Figure 7). The 11 metabolites shown in section O1/O2 and their trajectories (Figure 6) form a signature common to both donors, which we propose may potentially be used as an osteogenesis-specific (i.e., distinct from controls, dashed lines) and donor-independent marker (at least based on the 2 donors considered here). However, as for the suggested proliferation signature, this hypothesis requires verification in additional donors. In particular, this signature includes high-end values (day 21) of GSH, adenosine, uridine, Cho, Etn and unassigned at *δ* 3.48, lower end values of methylguanidine, PCr, inosine, PCho and intermediate end values of taurine. Changes in Cho, Etn, PCho, Cr and PCr result in higher Cr/PCr, Cho/PCho, Etn/PCho ratios, as characteristic of osteogenesis. It is worth mentioning that, although BCCAs, glycine and phenylalanine trajectories may not necessarily be distinct from controls for both donors (indeed, during osteodifferentiation these amino acid levels overlap with control trajectories for donor 2, Figure S6) and, therefore, not strictly osteogenic-specific, their levels also converged from early on (day 4 for BCAAs and phenylalanine, and day 7 for glycine, Figure S6) until day 21.

Finally, the proposed donor-independent metabolite variations identified above (specifically, (i) the 6 robust proliferation markers: alanine, aspartate, proline, tyrosine, ADP and ATP; (ii) the 11 proposed osteogenic-specific metabolites, found in section O1/O2 and in addition to the Cr/PCr, Cho/PCho, Etn/PCho ratios; and (iii) the 5 metabolites found to be osteogenic-specific at least for one of donors 1 and 2: BCAAs, glycine and phenylalanine) were sought for an independently chosen donor 3, in order to attempt some marker validation (ideally to be extended to a higher number of donors). Figure S10A indicates that 5 out of the 6 proliferation markers are confirmed to vary in similar manners (namely, alanine, aspartate, proline, tyrosine and ADP), thus excluding ATP as it varies differently in donor 3. Even though a control assay was not available for donor 3 cells, we suggest that the above subset of 5 metabolites may be promising markers of proliferation, and ATP levels possibly depending on other metabolic features. Regarding the proposed osteodifferentiation markers (Figure S10B), GSH and taurine levels converge after day 21, while those of adenosine, choline, ethanolamine and unassigned at δ 3.48 converge at day 14 (and diverge at day 21 for donor 3, compared to donors 1 and 2). Hence, GSH and taurine seem to be promising markers to be read at day 21, whereas adenosine, choline, ethanolamine and δ 3.48 appear as potential markers to be read at day 14 (the reason for their divergence at day 21 for donor 3 remain unclear at this stage, one possibility residing on the slight differences in the extraction procedure). Methylguanidine and PCr (and hence Cr/PCr) could not be evaluated due to signal overlap with aspartate resonances (due to a shift of methylguanidine peaks, possibly due to small pH changes) and creatinine resonances (due to the presence of this compound in donor 3, and its absence in donors 1 and 2), respectively. This suggests that if methylguanidine and PCr are to be confirmed/used as potential markers, their monitoring will depend closely on pH and creatinine content. Finally, inosine, uridine and PCho exhibited different trajectories for donor 3, compared to donors 1 and 2, suggesting that these 3 compounds do not seem to be part of a generalized donor-independent osteogenic signature. In spite of this, the Cho/PCho ratio showed a similar behaviour until day 14 for all 3 donors (unlike Etn/PCho), thus confirming Cho/PCho as a potential marker. Furthermore, the levels of BCAAs, phenylalanine and glycine were observed to converge after day 7 (Figure S10C), as observed for donors 1 and 2, which confirmed their potential as osteo-specific markers.

## 4. Discussion

This work has compared the detailed metabolic profiling of two randomly chosen donors, during cell proliferation alone and osteogenic differentiation. Donor 1 was found to be more osteogenic (higher ALP and total calcium levels) and slightly more proliferating (higher dsDNA levels in both basal and osteogenic conditions), compared to donor 2. As only two donors were considered, discussion of the reasons for these distinct characteristics is out of scope here, however, the metabolic information found may be correlated to the different donor biochemical parameters.

Cell proliferation in donor 1 is accompanied by a well-defined metabolic trajectory, which is not as clear in donor 2. However, amino acids generally tend to accumulate before day 4 in both donors (perhaps in preparation for use for protein synthesis) [36,61], is generally used up between days 7 and 14 (suggesting this period as particularly active regarding protein biosynthesis and anaplerotic feeding of the TCA cycle for energy production), and subsequently accumulating again until day 21. It becomes apparent that, in spite of their different cell proliferation degrees, hAMSCs are capable of regulating the levels of alanine, aspartate, glutamate, glycine, proline, tyrosine, methylguanidine and GSH (and perhaps lower abundance amino acids histidine, glutamine and threonine) to converging end levels. The levelling off of this group of amino acids and of the tripeptide GSH may, respectively, reflect an underlying equilibrium in protein metabolism and/or TCA activity, as well as in active antioxidative protection mechanisms (related to the noted general decrease in GSH). Energy metabolism (decreased ATP and ADP levels), nucleotide metabolism (fluctuating adenosine and NAD^+^ levels, increased inosine and decreased 1-MNA), and protein glycosylation (decreased UDP-GlcNAc) [36,62] also seem to exhibit end donor-independent levels, suggesting that these pathways may be effectively regulated to similar extents within the cells of different donors, over the 21-day period. Within the above proposed donor-independent proliferating characteristics, the general variations in alanine (fluctuation), aspartate (decrease), proline (decrease), tyrosine (increase), ATP and ADP (both decreasing), were maintained even in the presence of osteogenic differentiation, indicating that the biochemical roles of these four amino acids and the main energy metabolism traits related to ATP/ADP are particularly robust indicators of ongoing cell proliferation. This is particularly interesting for aspartate, suggested before [36] to vary due to its engagement in osteogenic-related protein synthesis, but here observed to depend mainly on cell proliferation. In addition, unaltered overlapping Cr/PCr, Cho/PCho and Etn/PCho ratios also characterize cell proliferation (in spite of the donor-dependent trajectory for the individual metabolites), which suggests some equilibrium characterizing creatine metabolism and membrane biosynthesis throughout the 21-day period, irrespective of cell donor characteristics. On the other hand, the different extents of cell proliferation and other aspects of donor variability (unaccounted for in this work) seem to be reflected in donor-dependent variations in TCA activity (anaplerotic amino acids: BCAAs and phenylalanine, and intermediate succinate), glycolysis (glucose), lactic fermentation (lactate) and lipid metabolism (acetate and glycerol).

Upon osteogenic cues, the most osteogenic donor (donor 1) exhibits a clearer metabolic switch from day 7 to day 14, the overall process being accompanied by generally more persisting changes in several amino acids. This indicated that a higher osteogenic differentiation extension is clearly reflected in amino acid metabolism, at least including their involvement in the biosynthesis of osteogenic protein markers, but also probably as substrates to feed into the TCA cycle. More extensive osteogenesis also leads to lower levels of creatine (either due to additional metabolic fates or less production through other pathways) and of the cell membrane precursors Cho and Etn. Indeed, the increases in Cr/PCr, Cho/PCho and Etn/PCho ratios identified before as markers of osteogenesis are not as pronounced for the more extensively osteogenic donor. In relation to Cho/PCho and Etn/PCho ratios, it is unclear at this stage if their diminished levels result from differentiation and/or proliferation mechanisms, as the more osteogenic donor 1 also shows slightly enhanced cell proliferation. Furthermore, choline levels are also known to depend on intra/extracellular cross-talk mechanisms [37], which may be influencing Cho/PCho ratios.

In spite of the above-discussed metabolic differences between the two donors, some osteogenic-specific metabolite changes could be advanced as donor-independent characteristics of osteogenic differentiation. These comprise increased GSH (oxidative protection), increased Cho and Etn and corresponding ratios to PCho (here possibly reflecting the observed depletion of PCho and PEtn as phosphate sources for mineralization [36,63,64]), increased adenosine and decreased inosine (purine metabolism), increased uridine (pyrimidine metabolism), and decreases in both methylguanidine (creatinine metabolism) [65] and PCr (again also supporting phosphate formation) [36]. As a last note, BCAAs, phenylalanine and glycine also converge into common levels at the end of osteogenesis, independently of their origin. These are, however, not strictly osteogenic-specific, as their evolution overlaps with control trajectories for donor 2, probably reflecting the less extent of osteogenesis and advancing that these amino acids may be indicative of the extent of osteogenesis, as suggested earlier. Clearly, the above considerations require validation in a larger number of donors and such was attempted here for a third donor, with distinct characteristics namely regarding donor age, harvesting procedure, cell passage (7, compared to 5 and 6 for donors 1 and 2) and extraction protocol. In spite of these differences, many of the potential marker metabolites could be confirmed, with the exception of ATP (as an indicator of proliferation) and adenosine, uridine and PCho (as indicators of osteogenesis), while methylguanidine and PCr evolutions could not be unambiguously confirmed due to signal overlap with other metabolites in the NMR spectra of donor 3. The latter observations indicate the importance of a careful pH adjustment protocol to avoid peak shifts that may lead to overlap with methylguanidine, and of monitoring PCr when in presence of higher creatinine overlapping peaks (e.g., using a method other than NMR).

## 5. Conclusions

This paper describes, for the first time to our knowledge, an untargeted NMR metabolomics study to measure hAMSCs polar endometabolome as a function of proliferation and osteogenic differentiation, to identify donor-dependent and donor-independent metabolic features of each process, firstly considering two donors. A set of 18 metabolites could be identified as a donor-independent marker of cell proliferation, including particularly robust variations in alanine, aspartate, proline, tyrosine, ATP and ADP, which are not affected by osteogenic differentiation. Regarding osteogenesis, a set of 11 metabolites were proposed as composing an osteogenic-specific donor-independent signature. This signature includes altered levels of taurine, PCr (and Cr/PCr ratios), GSH, methylguanidine, adenosine, inosine, uridine, Cho, Etn, PCho (and Cho/PCho and Etn/PCho ratios) and a still unassigned resonance at 3.48 ppm. In particular, some metabolites previously suggested to relate to osteogenesis were now linked preferably to the underlying cell proliferation process. Although the fact that only two donors were considered in detail is a clear limitation of this work, their distinct proliferation and osteogenic abilities enabled putative correlation between these properties and the metabolic adaptations to be advanced. Interestingly, many of the above signatures could be confirmed for a third donor, although the results reported here necessarily require validation in a larger cohort of donors, then also enabling a more robust correlation between the metabolic profile and (i) biochemical markers of proliferation and osteogenesis and (ii) donor characteristics. Future developments may entail the use of a validated metabolic signature of successful osteogenic differentiation to help monitor and guide the process into a pure lineage outcome. We further propose that such metabolic features may also potentially be used to predict the osteogenic ability of different donors/tissues at an early stage of the process, and even help evaluate in vivo post-implantation MSC performance.

## Data Availability

The spectral data obtained within this work will be made available on the Metabolomics Workbench website (https://www.metabolomicsworkbench.org/, accessed on 21 November 2022).

## Supplementary Materials

The following supporting information can be downloaded at: https://www.mdpi.com/article/10.3390/cells11233745/s1, Figure S1: Biochemical biomarkers of the osteogenic differentiation for donors 1 and 2, for hAMSCs cultures in basal/growth and osteogenic media; Figure S2: Multivariate analysis of full resolution ^1^H NMR spectra acquired for polar extracts of osteodifferentiating and proliferating hAMSCs, considering results from donors 1 and 2; Figure S3: Heatmap showing statistically significant metabolic variations during hAMSCs proliferation; Figure S4: Donor-dependent metabolic variations observed during hAMSCs proliferation; Figure S5: Heatmaps showing statistically significant metabolic variations during the osteodifferentiation and proliferation of hAMSCs from donors 1 and 2; Figure S6: Variations in amino acids in osteodifferentiating and proliferating hAMSCs from donors 1 and 2; Figure S7: Variations in amino acid derivatives and organic acids in osteodifferentiating and proliferating hAMSCs from donors 1 and 2; Figure S8: Variations in nucleotides and derivatives in osteodifferentiating and proliferating hAMSCs from donors 1 and 2; Figure S9: Variations in membrane precursors and other compounds in osteodifferentiating and proliferating hAMSCs from donors 1 and 2; Figure S10: Comparison of the proliferation and osteogenic markers proposed for donors 1 and 2 with a randomly chosen donor 3; Table S1: ^1^H NMR assignment of polar endometabolites identified in hAMSCs during osteodifferentiation or proliferation.
