# Supplementary material for: An Intracellular Metabolic Signature as a Potential Donor-Independent Marker of the Osteogenic Differentiation of Adipose Tissue Mesenchymal Stem Cells
Source: Cells. 2022 Nov 23;11(23):3745. doi: 10.3390/cells11233745 (PMC9739047; doi:10.3390/cells11233745)
Supplement: Supplementary file 1 [file cells-11-03745-s001.zip › cells-1965936-supplementary.pdf]

Figure S1

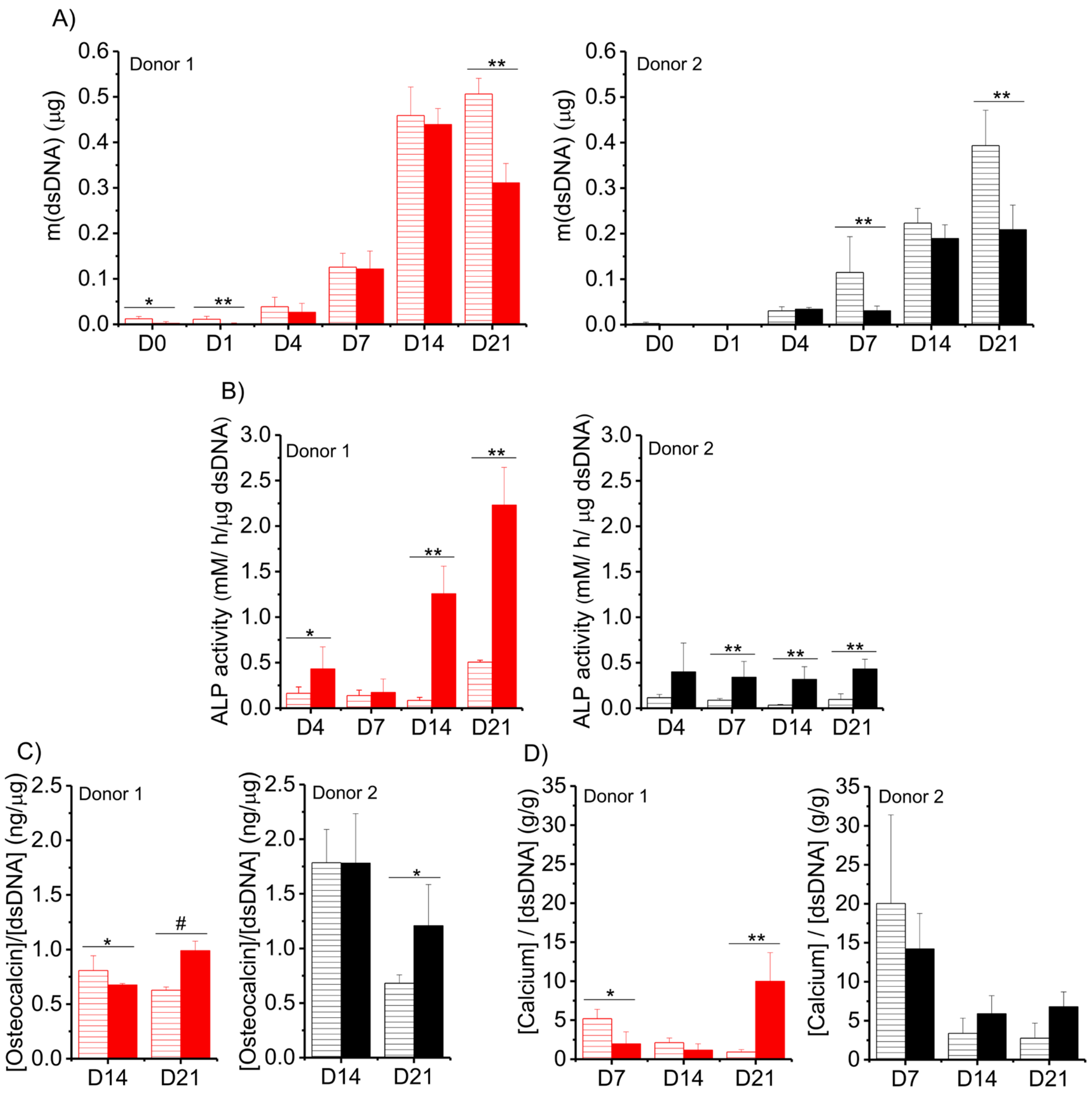
A)
Donor 1
m(dsDNA) (µg)
Donor 2
D0
D1
D4
D7
D14
D21
B)
ALP activity (mM/ h/µg dsDNA)
ALP activity (mM/ h/ µg dsDNA)
C)
[Osteocalcin]/[dsDNA] (ng/µg)
D)
[Calcium] / [dsDNA] (g/g)

Figure S2

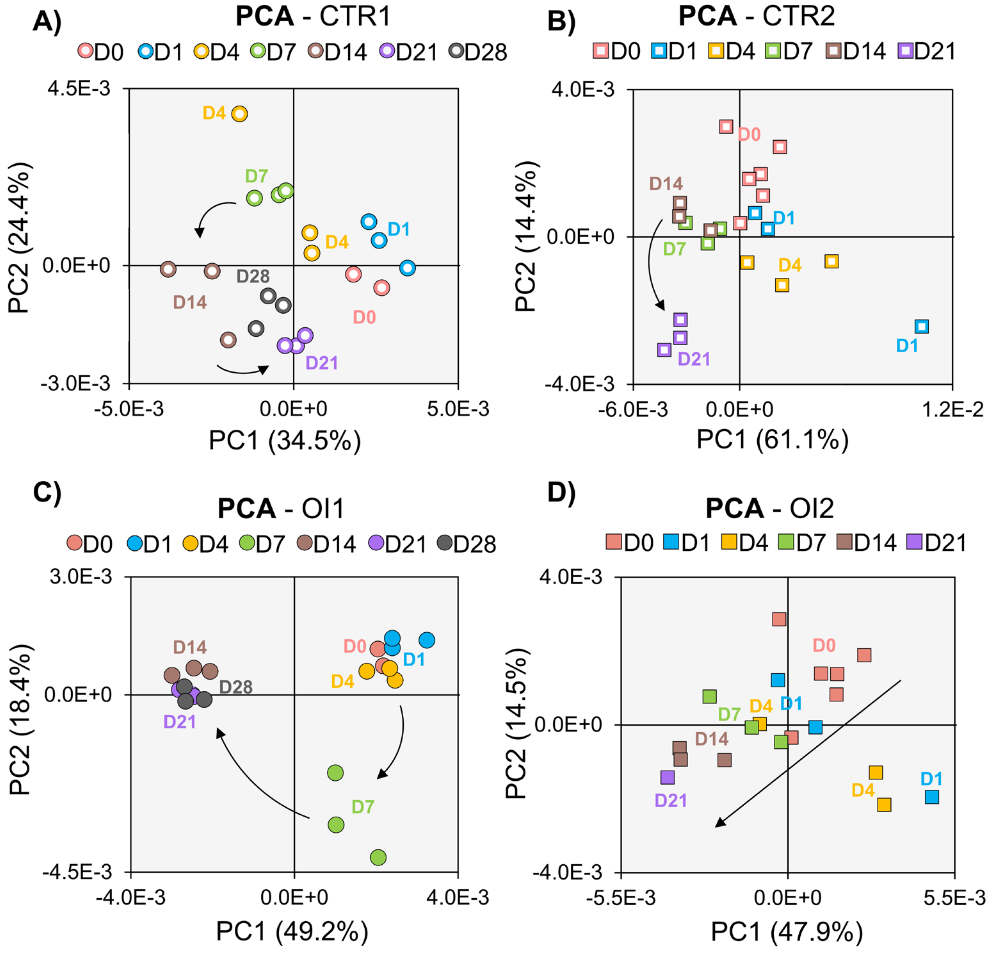

A)
PCA - CTR1
D0 D1 D4 D7 D14 D21 D28
4.5E-3
0.0E+0
-3.0E-3
-5.0E-3
0.0E+0
5.0E-3
PC2 (24.4%)
PC1 (34.5%)
B)
PCA - CTR2
D0 D1 D4 D7 D14 D21
4.0E-3
0.0E+0
-4.0E-3
-6.0E-3
0.0E+0
1.2E-2
PC2 (14.4%)
PC1 (61.1%)
C)
PCA - OI1
D0 D1 D4 D7 D14 D21 D28
3.0E-3
0.0E+0
-4.5E-3
-4.0E-3
0.0E+0
4.0E-3
PC2 (18.4%)
PC1 (49.2%)
D)
PCA - OI2
D0 D1 D4 D7 D14 D21
4.0E-3
0.0E+0
-4.0E-3
-5.5E-3
0.0E+0
5.5E-3
PC2 (14.5%)
PC1 (47.9%)

Figure S3

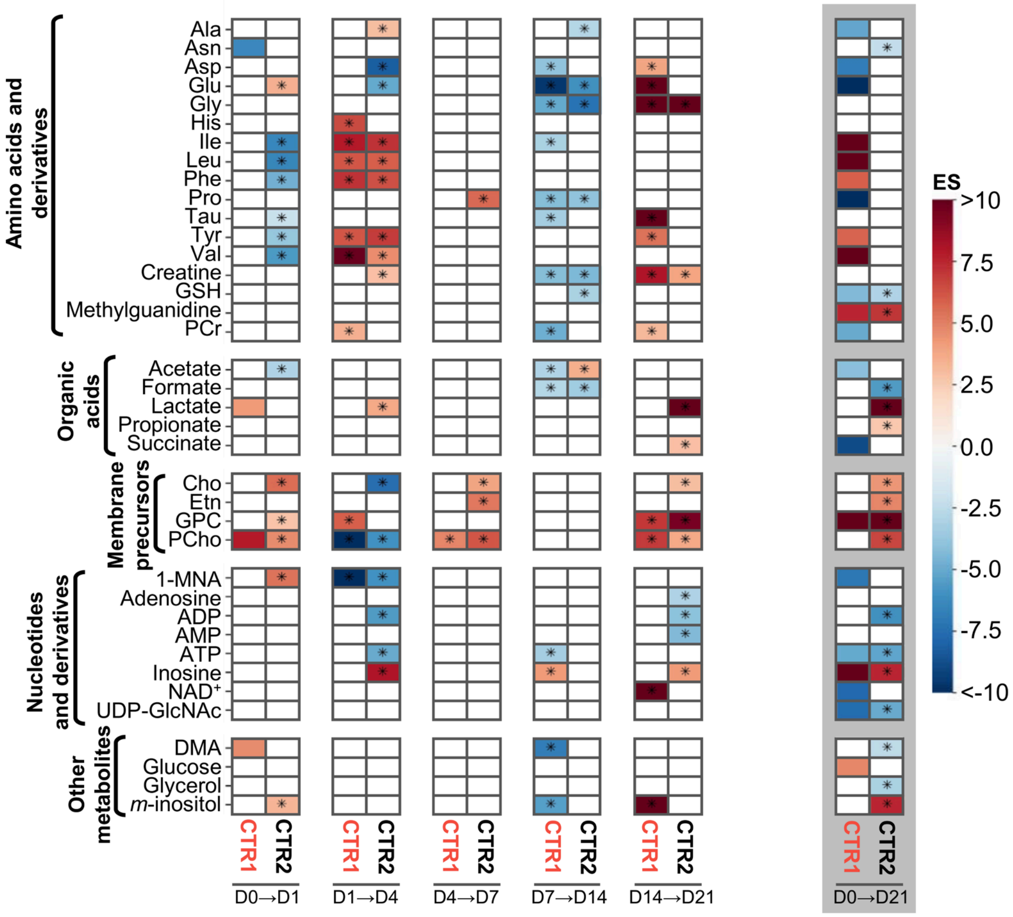
Amino acids and derivatives
Ala
Asn
Asp
Glu
Gly
His
Ile
Leu
Phe
Pro
Tau
Tyr
Val
Creatine
GSH
Methylguanidine
PCr
Organic acids
Acetate
Formate
Lactate
Propionate
Succinate
Membrane precursors
Cho
Etn
GPC
PCho
Nucleotides and derivatives
1-MNA
Adenosine
ADP
AMP
ATP
Inosine
NAD+
UDP-GlcNAc
Other metabolites
DMA
Glucose
Glycerol
m-inositol
CTR1
CTR2
D0→D1
D1→D4
D4→D7
D7→D14
D14→D21
D0→D21
ES
>10
7.5
5.0
2.5
0.0
-2.5
-5.0
-7.5
<-10

Figure S4

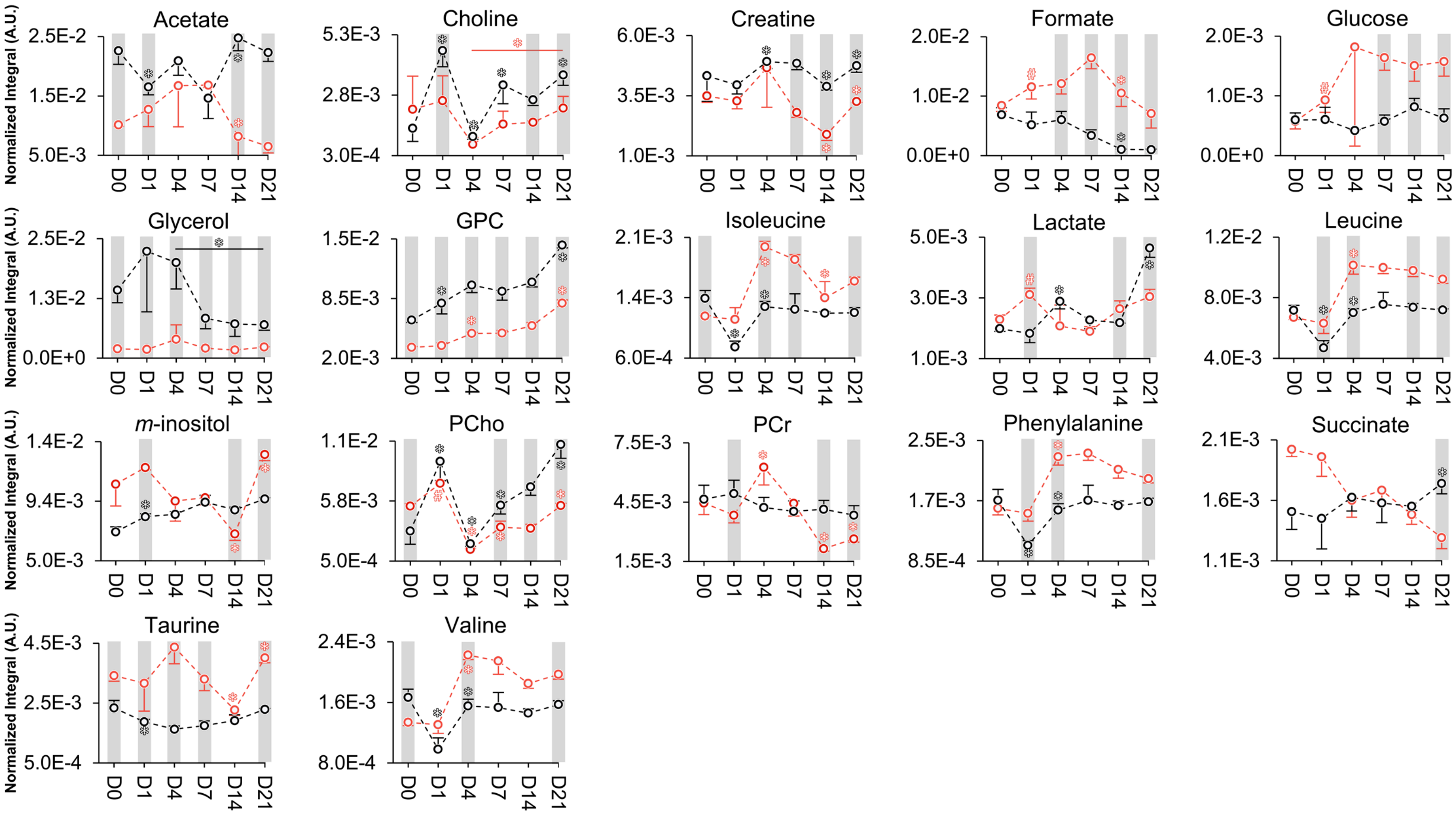
Acetate
Choline
Creatine
Formate
Glucose
Glycerol
GPC
Isoleucine
Lactate
Leucine
m-inositol
PCho
PCr
Phenylalanine
Succinate
Taurine
Valine
Normalized Integral (A.U.)
D0
D1
D4
D7
D14
D21
2.5E-2
1.5E-2
5.0E-3
5.3E-3
2.8E-3
3.0E-4
6.0E-3
3.5E-3
1.0E-3
2.0E-2
1.0E-2
0.0E+0
1.3E-2
1.5E-2
8.5E-3
2.0E-3
2.1E-3
1.4E-3
6.0E-4
5.0E-3
3.0E-3
1.0E-3
1.2E-2
8.0E-3
4.0E-3
1.4E-2
9.4E-3
1.1E-2
5.8E-3
7.5E-3
4.5E-3
1.5E-3
2.5E-3
1.7E-3
8.5E-4
1.6E-3
1.1E-3
2.5E-3
5.0E-4
2.4E-3
8.0E-4

Figure S5

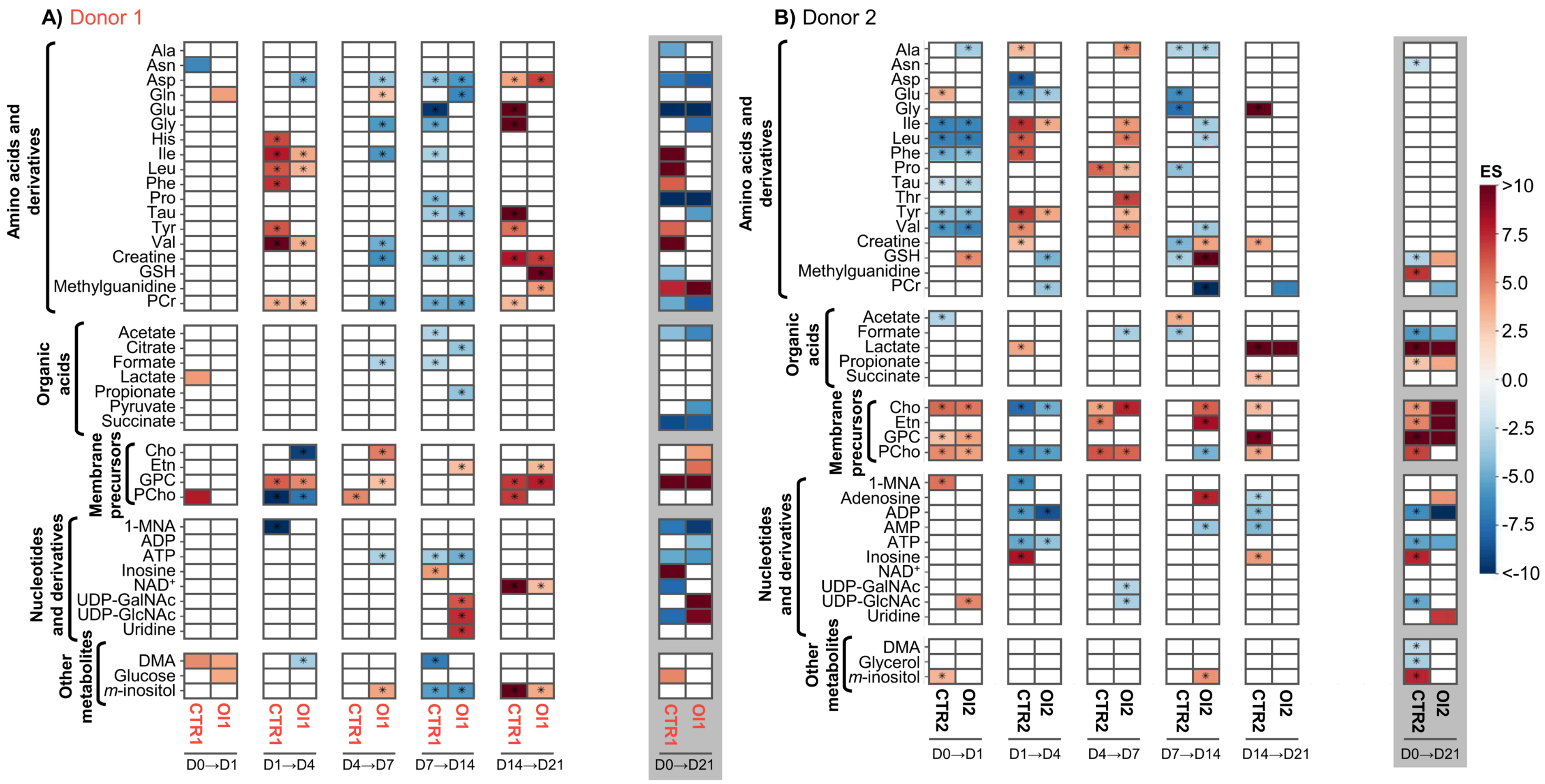

A) Donor 1
B) Donor 2
Amino acids and derivatives
Ala
Asn
Asp
Gln
Glu
Gly
His
Ile
Leu
Phe
Pro
Tau
Thr
Tyr
Val
Creatine
GSH
Methylguanidine
PCr
Organic acids
Acetate
Citrate
Formate
Lactate
Propionate
Pyruvate
Succinate
Membrane precursors
Cho
Etn
GPC
PCho
Nucleotides and derivatives
1-MNA
Adenosine
ADP
AMP
ATP
Inosine
NAD⁺
UDP-GalNAc
UDP-GlcNAc
Uridine
Other metabolites
DMA
Glucose
Glycerol
m-inositol
CTR1
OI1
CTR2
OI2
D0→D1
D1→D4
D4→D7
D7→D14
D14→D21
D0→D21
ES
>10
7.5
5.0
2.5
0.0
-2.5
-5.0
-7.5
<-10

Figure S6

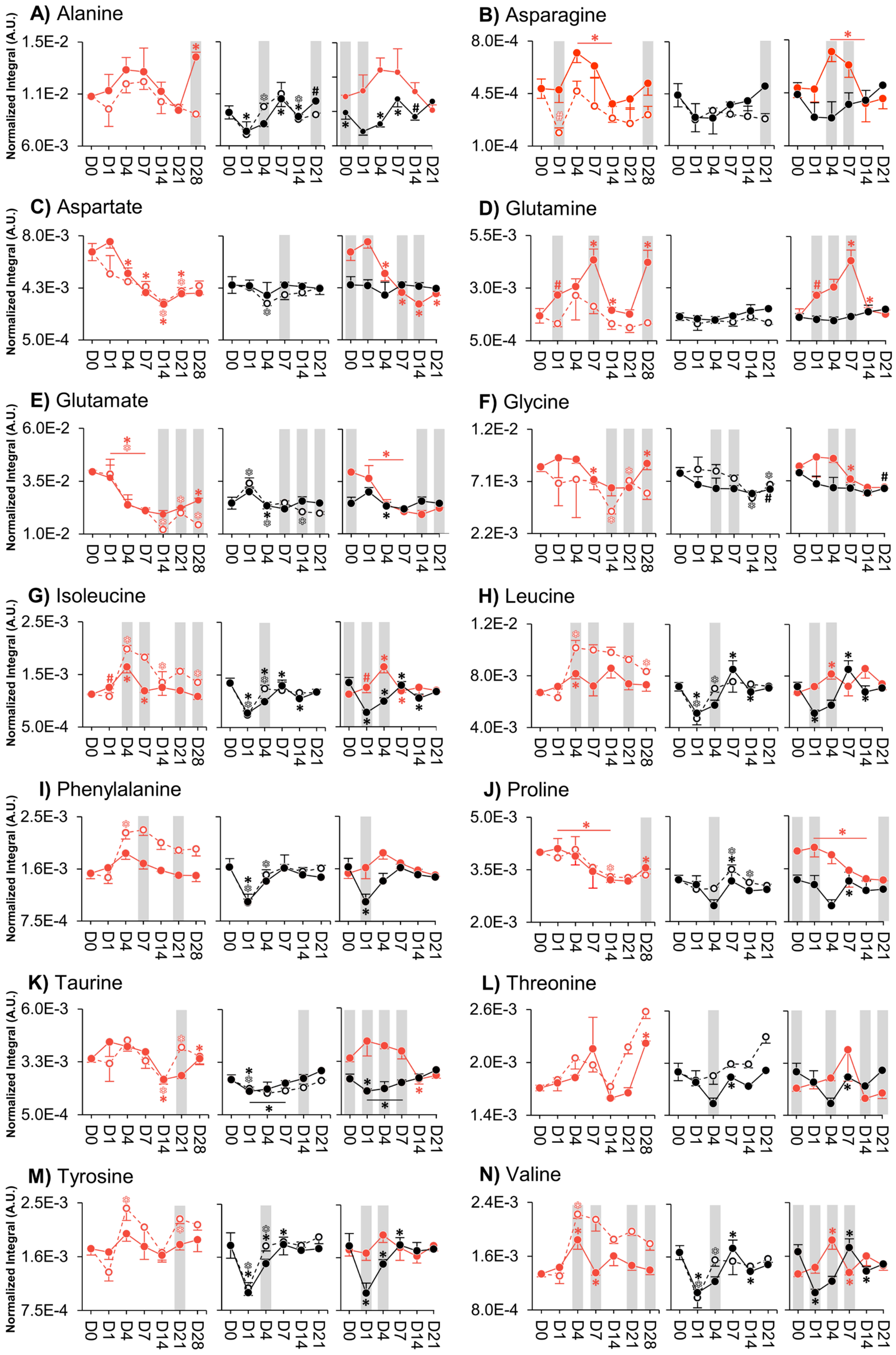
A) Alanine
B) Asparagine
C) Aspartate
D) Glutamine
E) Glutamate
F) Glycine
G) Isoleucine
H) Leucine
I) Phenylalanine
J) Proline
K) Taurine
L) Threonine
M) Tyrosine
N) Valine
Normalized Integral (A.U.)
1.5E-2
1.1E-2
6.0E-3
8.0E-4
4.5E-4
1.0E-4
8.0E-3
4.3E-3
5.0E-4
5.5E-3
3.0E-3
5.0E-4
6.0E-2
3.5E-2
1.0E-2
1.2E-2
7.1E-3
2.2E-3
2.5E-3
1.5E-3
5.0E-4
1.2E-2
8.0E-3
4.0E-3
2.5E-3
1.6E-3
7.5E-4
5.0E-3
3.5E-3
2.0E-3
6.0E-3
3.3E-3
5.0E-4
2.6E-3
2.0E-3
1.4E-3
2.5E-3
1.6E-3
7.5E-4
2.4E-3
1.6E-3
8.0E-4
D0
D1
D4
D7
D14
D21
D28

Figure S7

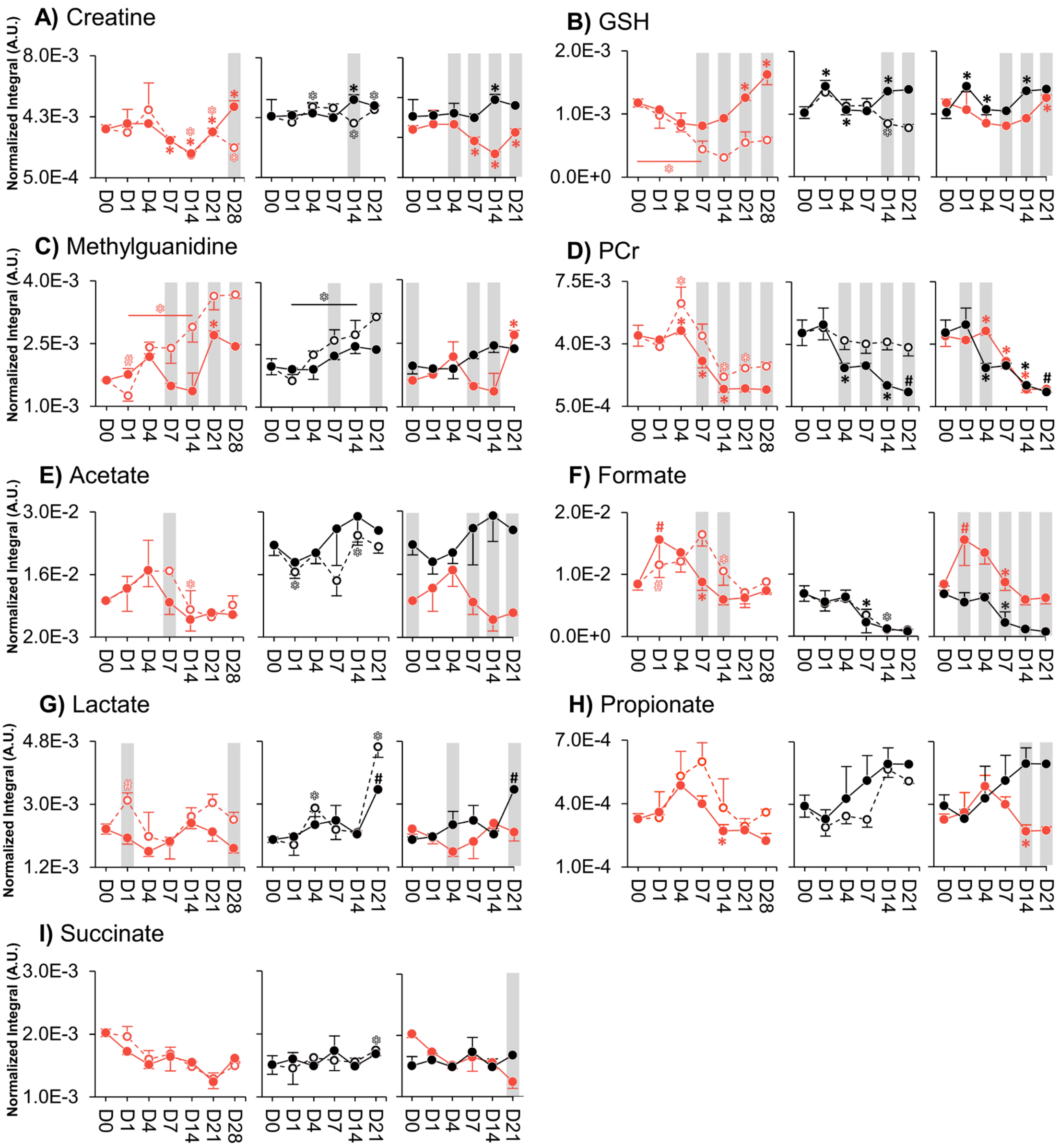
A) Creatine
B) GSH
C) Methylguanidine
D) PCr
E) Acetate
F) Formate
G) Lactate
H) Propionate
I) Succinate
Normalized Integral (A.U.)
8.0E-3
4.3E-3
5.0E-4
2.0E-3
1.0E-3
0.0E+0
4.0E-3
2.5E-3
1.0E-3
7.5E-3
4.0E-3
5.0E-4
3.0E-2
1.6E-2
2.0E-3
2.0E-2
1.0E-2
0.0E+0
4.8E-3
3.0E-3
1.2E-3
7.0E-4
4.0E-4
1.0E-4
3.0E-3
2.0E-3
1.0E-3
D0
D1
D4
D7
D14
D21
D28

Figure S8

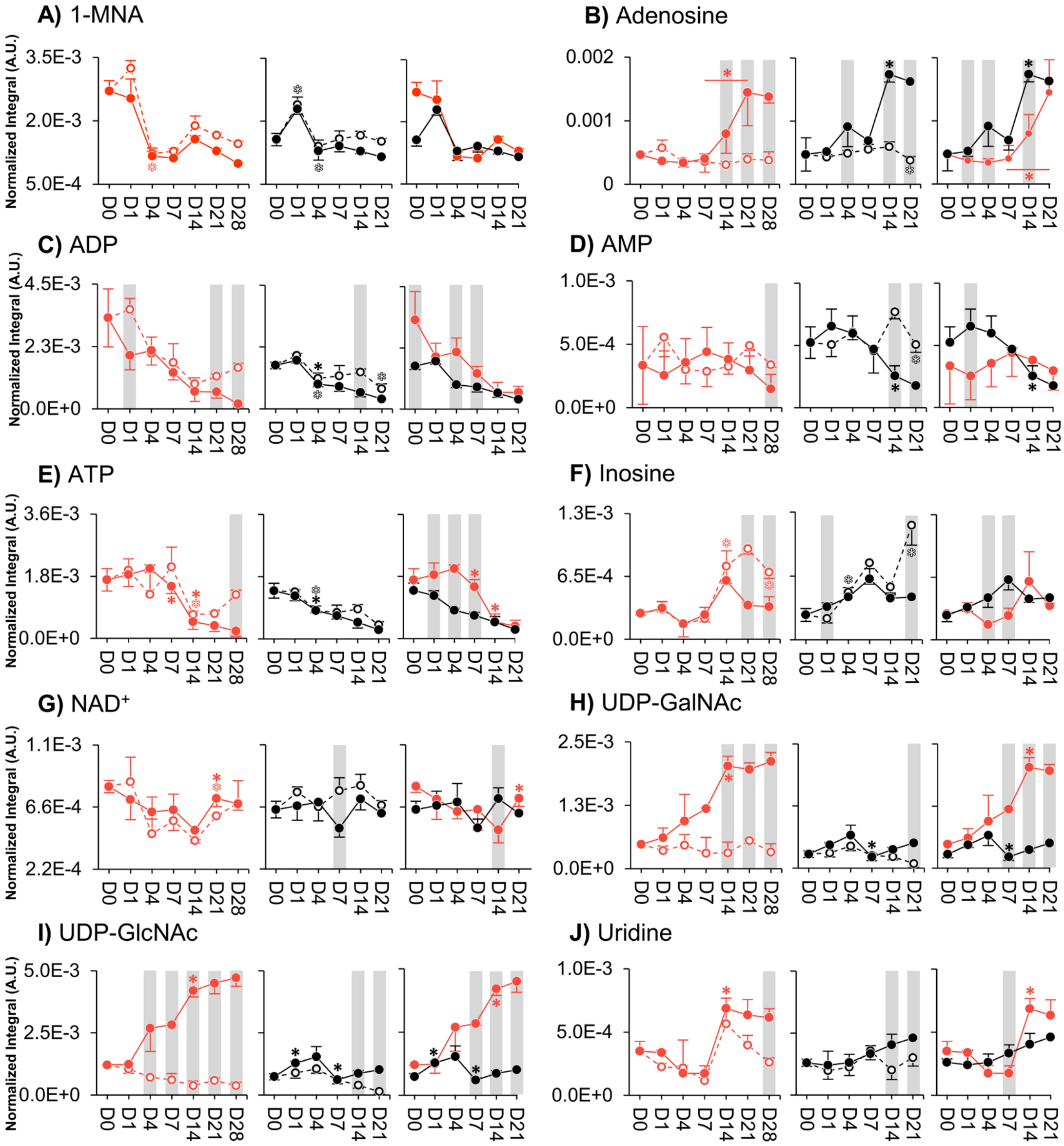

A) 1-MNA
B) Adenosine
C) ADP
D) AMP
E) ATP
F) Inosine
G) NAD+
H) UDP-GalNAc
I) UDP-GlcNAc
J) Uridine
Normalized Integral (A.U.)
D0
D1
D4
D7
D14
D21
D28

Figure S9

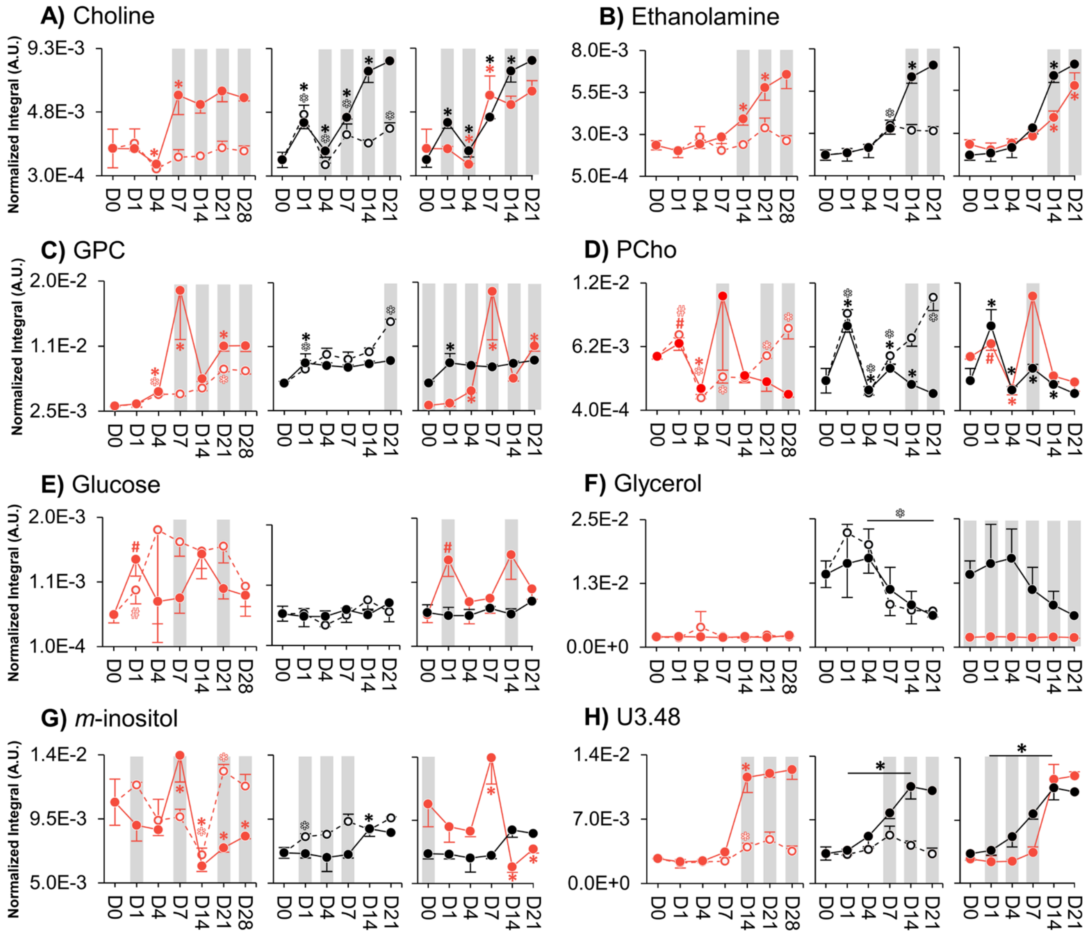

A) Choline
B) Ethanolamine
C) GPC
D) PCho
E) Glucose
F) Glycerol
G) *m*-inositol
H) U3.48
Normalized Integral (A.U.)
9.3E-3
4.8E-3
3.0E-4
8.0E-3
5.5E-3
3.0E-3
5.0E-4
2.0E-2
1.1E-2
2.5E-3
1.2E-2
6.2E-3
4.0E-4
2.0E-3
1.1E-3
1.0E-4
2.5E-2
1.3E-2
0.0E+0
1.4E-2
9.5E-3
5.0E-3
1.4E-2
7.0E-3
0.0E+0
D0
D1
D4
D7
D14
D21
D28

Figure S10

A) Proliferation markers

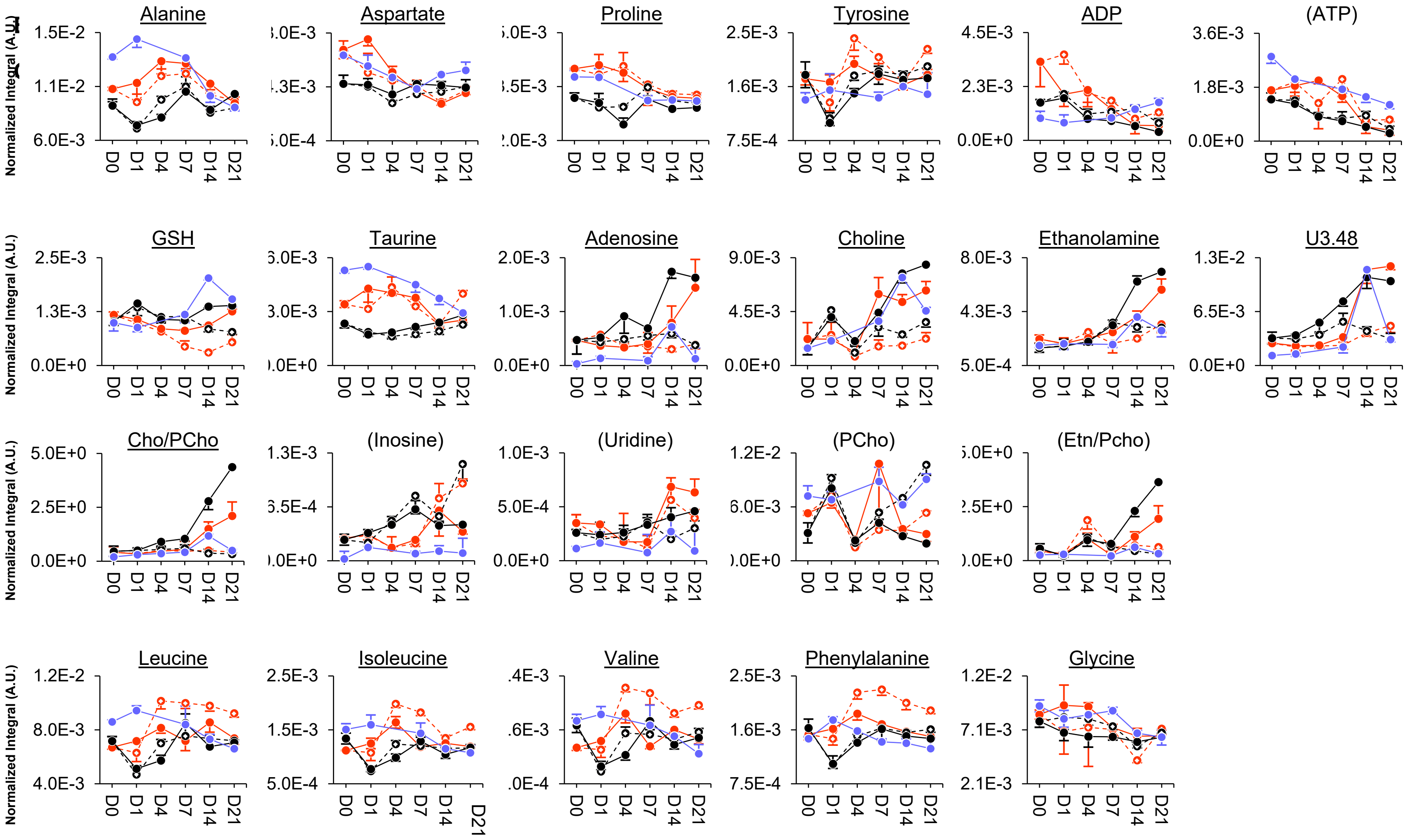

Table S1

Metabolite	**δ ^{1}H in ppm (multiplicity, assignment)**	**Donor 1** (OI1/CTR1)	**Donor 2** (OI2/CTR2)
1-MNA	4.49 (s, N-CH$_3$), 8.91 (d, 4-CH), 8.98 (d, 6-CH), 9.29 (s, 2-CH)	✓	#
Acetate	1.92 (s, *β*-CH$_3$)	✓	✓
Acetone	2.24 (s, *α*-CH$_3$)	✓	✓
Adenosine	3.84/3.92 (dd/dd, 5'-CH ribose), 4.30 (q, 4'-CH ribose), 4.44 (dd, 3'-CH ribose), 6.08 (d, 1'-CH ribose), 8.27 (s, 2-CH ring), 8.35 (s, 8-CH ring)	✓ (OI1) # (CTR1)	✓
ADP	4.24 (m, 5'-CH$_2$ ribose), 4.39 (m, 4'-CH ribose), 4.62 (m, 3'-CH ribose), 6.15 (d, 1'-CH ribose), 8.28 (s, 2-CH ring), 8.54 (s, 8-CH ring)	✓	✓
Alanine	1.48 (d, *β*-CH$_3$), 3.78 (q, *α*-CH)	✓	✓
AMP	4.02 (m, 5'-CH$_2$ ribose), 4.51 (m, 3'-CH ribose),6.15 (d, 1'-CH ribose), 8.28 (s, 2-CH ring), 8.62 (s, 8-CH ring)	✓	✓
Arginine	1.65 (m, ½ *γ*-CH$_2$), 1.72 (m, ½ *γ*-CH$_2$), 1.91 (m, *β*-CH$_2$), 3.24 (t, *δ*-CH$_2$)	✓	✓
Asparagine	2.86/2.95 (dd/dd, β-CH$_2$), 4.00 (dd, α-CH)	#	#
Aspartate	2.68/2.82 (dd/dd, *β*-CH$_2$), 3.90 (dd, *α*-CH)	✓	✓
ATP	4.22/4.29 (m/m, 5'-CH$_2$ ribose), 4.41 (m, 4'-CH ribose), 4.62 (m, 3'-CH ribose), 6.15 (d, 1'-CH ribose), 8.28 (s, 2-CH ring), 8.55 (s, 8-CH ring)	✓	✓
Choline	3.21 (s, N(CH$_3$)$_3$), 3.53 (m, N-CH$_2$), 4.07 (m, CH$_2$-OH)	✓	✓
Citrate	2.54 (d, *α*-CH & *β*-CH), 2.66 (d, *α'*-CH & *β'*-CH)	#	#
Creatine	3.04 (s, N-CH$_3$), 3.93 (s, N-CH$_2$)	✓	✓
Dimethylamine	2.73 (s, CH$_3$)	✓	✓
Ethanolamine	3.14 (t, CH$_2$-NH$_2$), 3.82 (t, CH$_2$-OH)	✓ (OI1) # (CTR1)	✓ (OI2) # (CTR2)
Formate	8.46 (s, HO-HC=O)	✓	✓
Glutamate	2.05/2.14 (m/m, *β*-CH$_2$), 2.35 (m, *γ*-CH$_2$), 3.76 (dd, *α*-CH)	✓	✓
Glutamine	2.14 (m, *β*-CH$_2$), 2.46 (m, *γ*-CH$_2$), 3.78 (t, *α*-CH)	✓ (OI1) # (CTR1)	#
Glutathione (reduced)	2.15 (m, *β*-CH$_2$ Glu), 2.55 (m, *γ*-CH$_2$ Glu), 2.96 (m, *β*-CH$_2$ Cys), 3.78 (m, *α*-CH Glu & *α*-CH$_2$, Gly), 4.57 (m, *α*-CH Cys)	✓	✓
**Glycerol**	3.56/3.66 (dd/dd, 1-CH$_2$ & 3-CH$_2$), 3.78 (m, 2-CH)	#	✓
Glycine	3.56 (s, α-CH$_2$)	✓	✓
GPC	3.24 (s, N(CH$_3$)$_3$)	✓	✓
Guanosine	5.99 (d, 1-CH ribose), 8.01 (s, 8-CH ring)	✓	✓
Histidine	7.08 (s, 5-CH ring), 7.84 (s, 2-CH)	#	#
Inosine	6.11 (d, 1'-CH ribose), 8.24 (s, 2-CH ring), 8.35 (s, 8-CH ring)	✓	✓
Isoleucine	0.94 (t, *δ*-CH$_3$), 1.02 (d, *γ'*-CH$_3$), 1.28/1.45 (m/m, *γ*-CH$_2$), 1.98 (m, *β*-CH), 3.66 (d, *α*-CH)	✓	✓
Lactate	1.33 (d, CH$_3$), 4.11 (q, CH)	✓	✓
Leucine	0.96 (d, *δ'*-CH$_3$), 0.97 (d, *δ*-CH$_3$), 1.72 (m, *γ*-CH & *β*-CH$_2$), 3.75 (m, *α*-CH)	✓	✓
Lysine	1.48 (m, *γ*-CH$_2$), 1.72 (m, *δ*-CH$_2$), 1.91 (m, *β*-CH$_2$), 3.03 (t, *ε*-CH$_2$, t), 3.77 (t, *α*-CH)	✓	✓
Methylguanidine	2.85 (s, CH$_3$)	✓	✓
myo-inositol	3.28 (t, 5-CH), 3.54 (dd, 1-CH & 3-CH), 3. 63 (t, 4-CH & 6-CH), 4.07 (t, 2-CH)	✓	✓
NAD$^+$	6.03 (d, 1'-CH ribose-adenine), 6.11 (d, 1'-CH ribose-	✓	✓

	nicotinamide), 8.18 (s, 2-C**H** adenine), 8.20 (m, 5-C**H** nicotinamide), 8.43 (s, 8-C**H** adenine), 8.84 (d, 4-C**H** nicotinamide), 9.15 (d, 6-C**H** nicotinamide), 9.34 (s, 2-C**H** nicotinamide)		
Pantothenate	0.90 (s, C**H**$_3$), 0.94 (s, C**H**$_3$), 2.42 (t, C**H**$_2$-COOH)	✓ (OI1)	✗
Phenylalanine	7.34 (m, 2-C**H** & 6-C**H** ring), 7.39 (m, 4-C**H** ring), 7.43 (m, 3-C**H** & 5-C**H** ring)	✓	✓
Phosphocholine	3.23 (s, N(C**H**$_3$)$_3$), 3.60 (m, N-C**H**$_2$), 4.17 (m, HPO_4^--C**H**$_2$)	✓	✓
Phosphocreatine	3.05 (s, N-C**H**$_3$), 3.95 (s, N-C**H**$_2$)	✓	✓
Proline	2.04 (m, *γ*-C**H**$_2$), 2.04/2.34(m/m, *β*-C**H**$_2$), 3.35/3.43 (dt/dt, *δ*-C**H**$_2$), 4.13 (dd, *α*-C**H**)	✓	✓
Propionate	1.06 (s, C**H**$_3$)	✓	✓
Propylene glycol	1.15 (d, C**H**$_3$)	✓	✓
Pyruvate	2.38 (s, C**H**$_3$)	#	#
Succinate	2.41 (s, C**H**$_2$)	✓	✓
Taurine	3.27 (t, S-C**H**$_2$), 3.42 (t, N-C**H**$_2$)	✓	✓
Threonine	1.33 (d, *γ*-C**H**$_3$), 3.59 (d, *β*-C**H**), 4.25 (dd, *α*-C**H**)	#	#
Tyrosine	6.91 (d, 3-C**H** & 5-**H** ring), 7.20 (d, 2-C**H** & 6-**H** ring)	✓	✓
UDP-GalNAc	5.55 (dd, 1''-C**H** galactose)	✓ (OI1)	#
UDP-GlcNAc	2.08 (s, C**H**$_3$ NAc), 3.82 (m, 3''-C**H** glucose & 6´´-C**H**$_2$ glucose), 3.88 (m, 6'' C**H**$_2$ glucose), 4.00 (m, 2''-C**H** glucose), 4.19/4.26 (m/m, 5'-C**H**$_2$ ribose), 4.30 (m, 4'-C**H** ribose), 4.38 (m, 2'-C**H** ribose & 3'-C**H** ribose), 5.52 (dd, 1''-C**H** glucose), 5.99 (m, 5-C**H** & 1'-C**H** ribose), 7.96 (d, 6-C**H**)	✓	✓
Uracil	5.81 (d, 5-C**H** ring), 7.55 (d, 6-C**H** ring)	# (OI1)	✗
Uridine	5.89 (d, 5-C**H** ring), 5.90 (d, 1-C**H** ribose), 7.88 (d, 6-C**H** ring)	✓	✓
Valine	1.00 (d, *γ'*-C**H**$_3$), 1.05 (d, *γ*-C**H**$_3$), 2.27 (m, *β*-C**H**), 3.62 (d, *α*-C**H**)	✓	✓
α-Glucose	5.24 (d, 1-C**H**)	✓	✓
β-Glucose	4.65 (d, 1-C**H**)	✓	✓

Captions to supplementary material

Figure S1. Biochemical biomarkers of the osteogenic differentiation for donor 1 (in black) and donor 2 (in red) for hAMSCs cultures in basal/growth (open bars with horizontal lines) or osteogenic (filled bars) media. (A) Quantification of double-stranded DNA (dsDNA) in lysed cells. (B) Alkaline phosphatase (ALP) activity measured after 4, 7, 14 and 21 days. (C) Quantification of osteocalcin after 14 and 21 days. (D) Calcium content measured after 7, 14 and 21 days. All parameters for osteogenic differentiation assessment (B, C and D) were normalized by dsDNA content. Data represent mean ± standard deviation (n = 6 for dsDNA, ALP and calcium; n = 3 for osteocalcin). For each timepoint, Wilcoxon Rank-sum statistical significance (*, p-values < 0.05; **, p-values < 0.01) between osteodifferentiated and undifferentiated cells is represented above the bars. Because day 21 from donor 1 has n < 3 samples, p-values < 0.09 (#) were also considered.

Figure S2. Multivariate analysis of full resolution ^{1}H NMR spectra acquired for polar extracts of osteodifferentiating (filled symbols) and proliferating hAMSCs (open symbols), considering results from donors 1 (dots) and 2 (squares). Scores scatter plots for principal component analysis (PCA) considering the stepwise time-course of proliferating hAMSCs (open symbols) from donors 1 (A) and 2 (B), as well as osteodifferentiating hAMSCs (filled symbols) from the same donors 1 (C) and 2 (D). Abbreviations: CTRi, proliferating (control) hAMSCs from donor *i*; Di, day i; OIi, osteoinduced hAMSCs from donor i; PC, principal component.

Figure S3. Heatmap showing statistically significant metabolic variations (|ES| > 0.50, ES error < 80%, Wilcoxon rank-sum p-value < 0.05) during hAMSCs proliferation, shown only in cases where spectral confirmation was observed. Rows correspond to metabolites and columns allow comparisons over time and between extreme days 0 and 21 (grey box), for both donors. The color scale varies from minimum (dark blue) to maximum (dark red) effect size (ES) values. Abbreviations: three-letter code used for amino acids; 1-MNA, 1-methylnicotinamide; ADP, adenosine diphosphate; AMP, adenosine monophosphate; ATP, adenosine triphosphate; Cho, choline; CTRi, proliferating (control) hAMSCs from donor i; Di, day i; DMA, dimethylamine; Etn, ethanolamine; GPC, glycerophosphocholine; GSH, glutathione (reduced); NAD^{+}, nicotinamide adenine dinucleotide; PCho, phosphocholine; PCr, phosphocreatine; UDP-GlcNAc, uridine diphospho-*N*-acetylglucosamine. Symbols: *, Wilcoxon Rank-sum test p-value < 0.05 compared to the previous timepoint (shown only in cases where spectral confirmation was observed). Comparisons without an asterisk (*) were selected exclusively based on visual inspection because day 0 from donor 1 has n < 3 samples.

Figure S4. Donor-dependent metabolic variations observed during hAMSCs proliferation. In alphabetical order of metabolite name, the graphs display normalized peak integrals for donor 1 (dashed black lines) and 2 (dashed red lines), for metabolites that vary differently throughout the 21 days or diverge at the end. Abbreviations: GPC, glycerophosphocholine; PCho, phosphocholine; PCr, phosphocreatine. Symbols (represented in the same colour as the corresponding line): *, Wilcoxon Rank-sum test p-value < 0.05 compared to the previous timepoint (shown only in cases where spectral confirmation was observed); #, relevant variations compared to the previous timepoint exclusively based on visual inspection (applicable when n < 3 samples, i.e. day 0 for donor 1). Grey bars highlight statistically significant differences (Wilcoxon Rank-sum p-value < 0.05) for each timepoint between donors (except for day 0, in which the grey bars refer to relevant variations exclusively based on visual inspection because n = 2 for donor 1).

Figure S5. Heatmaps showing statistically significant metabolic variations (|ES| > 0.50, ES error < 80%, Wilcoxon rank-sum p-value < 0.05) during the osteodifferentiation and proliferation of hAMSCs from (A) donor 1 and (B) donor 2, shown only in cases where spectral confirmation was observed. Rows correspond to metabolites and columns allow comparison over time and between days 0 and 21 (grey box). The color scale varies from minimum (dark blue) to maximum (dark red) effect size (ES) values. Abbreviations: OIi, osteoinduced hAMSCs from donor i; UDP-GalNAc, uridine diphospho-*N*-acetylgalactosamine; other metabolites abbreviated as shown in the caption of Figure S3. *, Wilcoxon Rank-sum test p-value < 0.05 compared to

the previous timepoint (shown only in cases where spectral confirmation was observed). Comparisons without an asterisk (*) were selected exclusively based on visual inspection because day 0 from donor 1 and day 21 from donor 2 have n < 3 samples.

Figure S6. Variations in amino acids in osteodifferentiating (solid lines) and proliferating hAMSCs (dashed lines) from donors 1 (in red) and 2 (in black). Results for each metabolite are shown in a set of 3 graphs of normalized integrals with the same scale, allowing for comparison of osteodifferentiating and proliferating hAMSCs from donors 1 (left) and 2 (middle), as well as the osteodifferentiation of both donors (right). Note that only the left panels (donor 1) show the trajectories throughout 28 days. Except for histidine (not shown due to low signal-to-noise ratio), all amino acids that varied significantly in at least one of the conditions/timepoints are shown (the apparent variation of threonine after day 7 is caused by signal overlapping). Abbreviations: three-letter code used for amino acids; Di, day i. *, Wilcoxon Rank-sum test p-value < 0.05 compared to the previous timepoint (shown only in cases where spectral confirmation was observed); #, relevant variations compared to the previous timepoint exclusively based on visual inspection (applicable when n < 3 samples, i.e. day 0 from donor 1 and osteogenic day 21 from donor 2). All symbols are represented in the same colour as the corresponding line, with open and filled symbols for proliferating and differentiating cells, respectively. Grey bars highlight statistically significant differences (Wilcoxon Rank-sum p-value < 0.05) for each timepoint between lines (except for day 0 in the middle panel and for day 21 in the middle and right panels, in which the grey bars refer to relevant variations exclusively based on visual inspection because n < 3 in day 0 from donor 1 and osteogenic day 21 from donor 2).

Figure S7. Variations in amino acid derivatives and organic acids in osteodifferentiating (solid lines) and proliferating hAMSCs (dashed lines) from donors 1 (in red) and 2 (in black). Graphs formatted as in Figure S6. GSH, glutathione (reduced); PCr, phosphocreatine.

Figure S8. Variations in nucleotides and derivatives in osteodifferentiating (solid lines) and proliferating hAMSCs (dashed lines) from donors 1 (in red) and 2 (in black). Graphs formatted as in Figure S6. 1-MNA, 1-methylnicotinamide; ADP, adenosine diphosphate; AMP, adenosine monophosphate; ATP, adenosine triphosphate; Di, day i; NAD^+, nicotinamide adenine dinucleotide; UDP-GalNAc, uridine diphospho-*N*-acetylgalactosamine; UDP-GlcNAc, uridine diphospho-*N*-acetylglucosamine.

Figure S9. Variations in membrane precursors and other compounds in osteodifferentiating (solid lines) and proliferating hAMSCs (dashed lines) from donors 1 (in red) and 2 (in black). Graphs formatted as in Figure S6. GPC, glycerophosphocholine; PCho, phosphocholine; Uδ, unassigned signal at chemical shift δ.

Figure S10. Comparison of the proliferation and osteogenic markers proposed for donors 1 (in red) and 2 (in black) with a randomly chosen donor 3 (in blue). Metabolite variations for osteodifferentiating and proliferating hAMSCs are represented by solid lines and dashed lines, respectively. The underlined metabolites (or ratios) correspond to those that varied similarly in all donors (at least at later stages) and thus were confirmed as (A) proliferation markers or osteogenic markers, either (B) osteogenic-specific or (C) partially osteogenic-specific (in case at least one donor behaves differently for osteogenesis and control). Metabolite names in brackets could not be confirmed as markers in donor 3, in addition to methylguanidine and creatine (not shown), which could not be monitored due to signal overlap. ADP, adenosine diphosphate; ATP, adenosine triphosphate; Cho, choline; Di, day i; Etn, ethanolamine; GSH, glutathione (reduced); PCho, phosphocholine; Uδ, unassigned signal at chemical shift δ.

Table S1. ^{1}H NMR assignment of polar endometabolites identified in hAMSCs during osteodifferentiation or proliferation. In the right two columns, the metabolites that are clearly present in at least one timepoint (✓), or that peaks near the noise level (#), or that are absent in all timepoints (Ⅹ), are indicated for each donor. Unless specified in brackets, each symbol (✓, # or Ⅹ) considers both proliferating and osteodifferentiating conditions. Abbreviations: 1-MNA, 1-methyl-nicotinamide; ADP, Adenosine diphosphate; AMP, adenosine monophosphate; ATP, adenosine triphosphate; CTRi, proliferating (control) hAMSCs from donor i; GPC,

glycerophosphocholine; NAD^+, nicotinamide adenine dinucleotide (oxidized); OIi, osteoinduced hAMSCs from donor i; UDP-GalNAc, uridine diphospho-*N*-acetylgalactosamine; UDP-GlcNAc, uridine diphospho-*N*-acetylglucosamine. Multiplicity: s, singlet; d, doublet; dd, doublet of doublets; dt, doublet of triplets; t, triplet; q, quartet; m, multiplet.
